# Potential role of host-derived quorum quenching in modulating bacterial colonization in the moon jellyfish *Aurelia aurita*

**DOI:** 10.1038/s41598-018-37321-z

**Published:** 2019-01-10

**Authors:** Nancy Weiland-Bräuer, Martin A. Fischer, Nicole Pinnow, Ruth A. Schmitz

**Affiliations:** Institute of General Microbiology, Christian-Albrechts University Kiel, Am Botanischen Garten 1-9, 24118 Kiel, Germany

## Abstract

Multicellular organisms can be regarded as metaorganisms, comprising of a macroscopic host interacting with associated microorganisms. Within this alliance, the host has to ensure attracting beneficial bacteria and defending against pathogens to establish and maintain a healthy homeostasis. Here, we obtained several lines of evidence arguing that *Aurelia aurita* uses interference with bacterial quorum sensing (QS) - quorum quenching (QQ) - as one host defense mechanism. Three *A*. *aurita*-derived proteins interfering with bacterial QS were identified by functionally screening a metagenomic library constructed from medusa-derived mucus. Native expression patterns of these host open reading frames (ORFs) differed in the diverse life stages (associated with different microbiota) pointing to a specific role in establishing the developmental stage-specific microbiota. Highly increased expression of all QQ-ORFs in germ-free animals further indicates their impact on the microbiota. Moreover, incubation of native animals with pathogenic bacteria induced expression of the identified QQ-ORFs arguing for a host defense strategy against confronting bacteria by interference with bacterial QS. In agreement, immobilized recombinant QQ proteins induced restructuring of polyp-associated microbiota through changing abundance and operational taxonomic unit composition. Thus, we hypothesize that additional to the immune system host-derived QQ-activities potentially control bacterial colonization.

## Introduction

**Figure 1:**
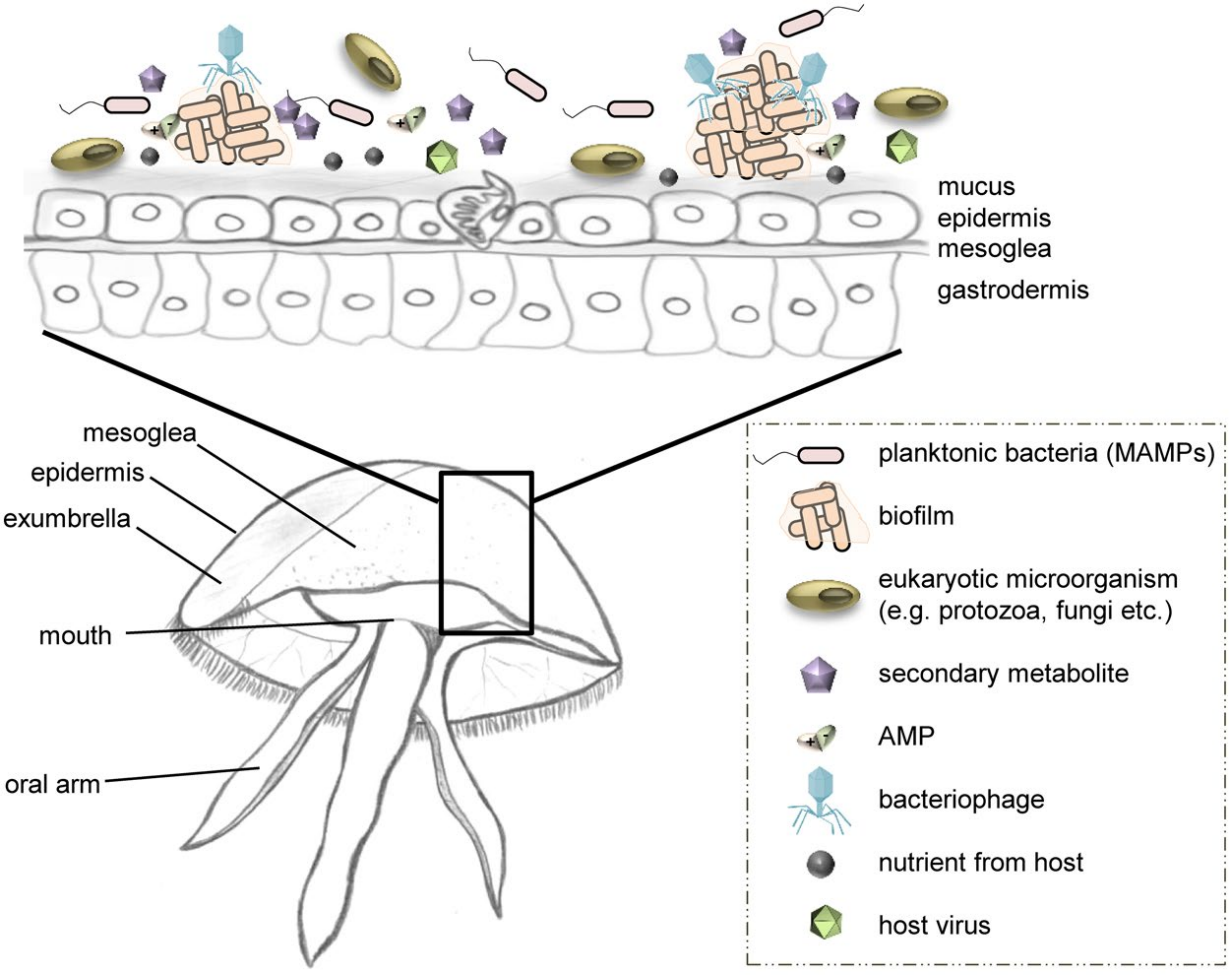
The metaorganism *Aurelia aurita*. Schematic view of the jellyfish metaorganism (*A*. *aurita* medusa in a cutaway side view) and selected factors that might influence bacterial colonization of the host surfaces. Drawings have been created by first author Weiland-Bräuer.

In natural habitats, members of different kingdoms of life do not exist isolated from one another but form complex associations and are connected to each other by diverse interactions^1^. Thus, every multicellular organism can be regarded as metaorganism comprising of the macroscopic host and its associated microorganisms (i.e. bacteria, archaea, fungi, protozoa, viruses)^2^ (Fig. 1, for more details see^3^). One large and evolutionary old environment, where host-microbe interactions evolved under unique conditions and various environmental pressures, is the ocean^4^. The ocean not only holds potential for diverse associations, but also puts a significant selective pressure on marine animals and plants entailing the co-evolution of multicellular organisms and associated microorganisms^5^. It is already known that epi- and endobiotic microbes offer an important protective effect for the host against various potentially harmful microorganisms and environmental changes^6^. Additionally, a healthy host has to continuously modulate and control its colonization through a range of different measures, e.g. by secretion of bioactive compounds (i.e. synthesized as part of the innate immune system, secondary metabolites), to establish and maintain a healthy metaorganismal homeostasis^7^.

An important and fundamental mechanism of bacteria regulating cell coordination of populations is bacterial cell-cell communication, so called quorum sensing (QS). QS is mediated through small molecules to control cellular processes in response to population density such as biofilm formation, colonization of a host, symbiosis, pathogenesis and virulence^8^. By interfering with QS, those coordinated behaviors can be influenced. One of the best-studied marine examples with regard to chemically mediated interactions between a host and its epiphytic bacteria is the red alga *Delisea pulchra*^9^. *D*. *pulchra* produces a halogenated furanone that antagonizes acylated homoserine lactones (AHL), the key signaling molecules of Gram-negative bacteria for QS. The presence of this antagonist inhibits biofilm formation, ultimately protecting the alga against fouling^10^. Consequently, fouling and disease can be controlled by a host-generated QS-interfering compound. Similar strategies against potential pathogens are suggested for a range of other marine multicellular organisms^11^.

In this study, the moon jellyfish *Aurelia aurita*, a member of the marine evolutionarily ancient phylum Cnidaria, has been put into focus. This invertebrate represents one of the most widely distributed Scyphozoa^12^ and shows a complex life cycle^13^. Here, after sexual reproduction, *A*. *aurita* releases planula larvae, which settle on a suitable substratum and develop to the sessile, benthic polyp stage. During strobilation, induced by environmental triggers, polyps form ephyrae through constriction of body parts, which are released and further develop to mature pelagic medusae (Fig. S1). The moon jelly offers a very simple morphology with only two tissue layers as epithelial barriers^14^. Invertebrates possess an innate immune system (IIS) with mucus-covered epithelia as a very first line of defense. Antibacterial compounds often found within the mucus enforcing this first barrier^15^. Additionally, receptors of the IIS recognize conserved molecules derived from bacteria such as flagellin, peptidoglycan and lipopolysaccharide (microorganism-associated molecular patterns, MAMPs)^16^. Besides an ancient IIS, interference with QS (Quorum quenching, QQ) might represent an additional strategy, potentially conserved from basal metazoans to vertebrates, to maintain homeostasis. Indications have been obtained for *Delisea pulchra*^17^, *Hydra vulgaris*^18^, and humans^19^. In contrast to a variety of marine species, very little is known about *Aurelia*-associated microorganisms or particular about the host-microbe interactions. Only recently, we reported on life stage-specific bacterial community patterns for *A*. *aurita*, which undergo significant restructuring during the course of metamorphosis^20^, arguing that the specifically associated microbiota might play important functional roles, for instance during *A*. *auritas* life cycle.

In the present study, we aimed to identify and analyze QQ activities of the host *A*. *aurita* interfering with bacterial cell-cell communication. Ultimately, we intended to gain insights into fundamental host-microbe interactions already present in a basal metazoan.

## Results

### Identification of three *A. aurita* derived proteins interfering with bacterial cell-cell-communication

A metagenomic fosmid library was constructed using mucus-extracted DNA from a single *A*. *aurita* medusa containing eukaryotic epithelial DNA and bacterial DNA of associated microbes. The large insert library comprises in total approximately 347 Mb metagenomic DNA present in over 9,200 fosmid clones allowing effective transcription of foreign DNA in the *E*. *coli* background. Cell-free cell extracts and culture supernatants of those fosmid clones were analyzed regarding their QS interfering activities using recently established *Escherichia coli* based reporter strains AI1-QQ.1 and AI2-QQ.1^21^. The functional screen resulted in the identification of 17 single fosmid clones interfering with Gram-negative acyl-homoserine lactones (AHLs), three interfering with universal autoinducer-2 (AI-2), and two fosmid clones simultaneously interfering with both QS signaling molecules.

**Figure 2:**
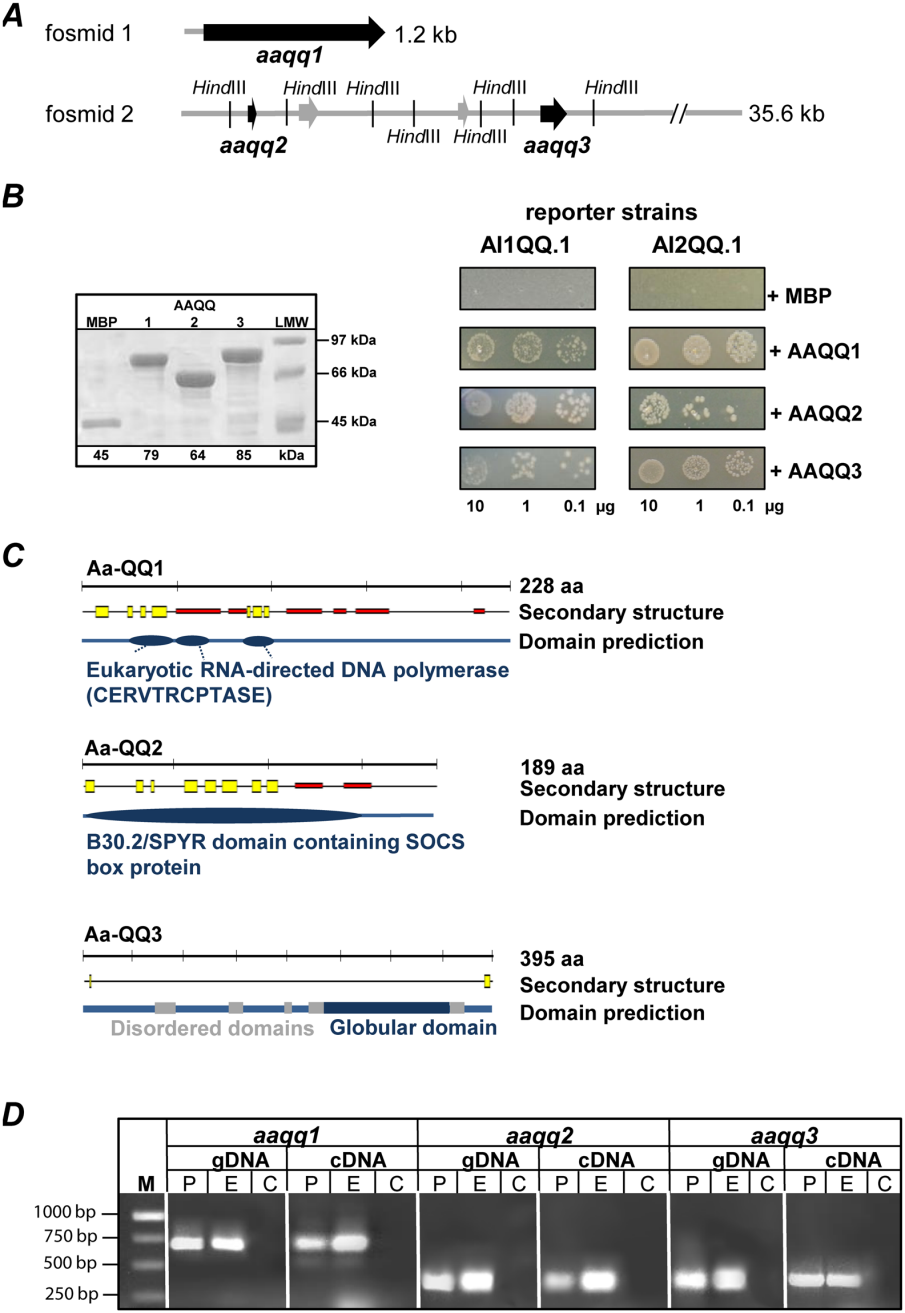
Characterization of *A*. *aurita* QQ ORFs. (**A**) Arrangements of predicted ORFs on original metagenomic fosmid inserts (supplemental dataset) are depicted, carrying the genes *aaqq1*, *aaqq2* and *aaqq3* linked to QQ phenotypes (black arrows, Accession Nos. JX274296-JX274298). (**B**) QQ-ORFs were cloned into pMAL-c2X and purified as MBP-fusion proteins by affinity chromatography. 5 µg of elution fractions were analyzed on 12% SDS-PAGE. Purified MBP-QQ proteins (0.1 µg, 1 µg and 10 µg) were tested regarding their QQ activities using reporter strains AI1-QQ.1 and AI2-QQ.1 (AHL, left panel; AI-2, right panel) monitoring QQ activities by growth. (**C**) QQ proteins were analyzed using secondary structure prediction software PredictProtein (http://www.predictprotein.org/) with following visual output in Profsec: yellow, strand; red, helix. InterProScan (http://www.ebi.ac.uk/Tools/pfa/iprscan/) and GlobPlot (http://globplot.embl.de/) were used for functional protein analysis. (**D**) Fragments of QQ-ORFs were PCR-amplified from genomic DNA as well as cDNA transcribed from RNA of antibiotic-treated polyps (P) and ephyrae (E). C, non-template control. The presented gel grouping is cropped from different gels.

The two fosmid clones conferring simultaneous interference with AHL and AI-2 QS were selected for further molecular characterization since the initial restriction pattern and sequence analysis revealed the presence of a 1.2 kb and a 35.6 kb eukaryotic DNA insert, respectively (Fig. 2). Based on sequence alignments using the NCBI database, a 0.7 kb ORF was identified on the 1.2 kb insert and designated *aaqq1*. A combined approach of FLX sequencing of the whole fosmid insert 2 and subcloning followed by QQ activity and sequence analysis resulted in identification of two putative QQ-ORFs *aaqq*2 and *aaqq3* exclusively responsible for the QQ activity besides two other ORFs conferring no QQ activity (Fig. 2A). Those three putative QQ-ORFs were cloned into pMALc2X and purified as MBP-fusion proteins for further characterization (Fig. S2). Subsequently, QQ activities against both signaling molecules were evaluated using reporter strains AI1-QQ.1 and AI2-QQ.1^21^ and respective control experiments (see also Materials and Methods), both clearly demonstrated that all three proteins effectively interfered with AHL and AI-2 QS signals (Fig. 2B). The QQ activity of all three purified proteins was further verified by demonstrating their biofilm inhibiting activities on static biofilms and *Klebsiella oxytoca* in flow cells (Fig. S3). Sequence-based protein domain prediction using PredictProtein (www.predictprotein.org/) and InterProScan (www.ebi.ac.uk/interpro/search/sequence-search) revealed that the secondary structure of AAQQ1 (228 aa, 25.1 kDa) consists of alternating β-sheets and α-helices forming predicted domains of eukaryotic RNA-directed DNA polymerase. For AAQQ2 (189 aa, 20.9 kDa) a SPYR domain was identified, an evolutionary ancient domain hypothesized as a component of immune defense^22^, whereas AAQQ3 (395 aa, 43.5 kDa) contains disordered and globular domains and is referred to as naturally unfolded protein of yet unknown function (Fig. 2C and Table S1). In addition, none of the QQ-ORF sequences neither contained putative signaling peptides according to SignalP 4.1 (http://www.cbs.dtu.dk/services/SignalP/) nor transmembrane domains according to TMHMM Server v. 2.0 (http://www.cbs.dtu.dk/services/TMHMM/). Generally, those sequenced based predictions neither allowed identifying the potential function or catalytic activity of the proteins nor gave insights into possible biochemical reaction(s) underlying the obtained QQ activity.

Further analysis using both, the *compagen* EST library (www.compagen.org/aurelia/) and the recently published six life stages comprising transcriptome of *A*. *aurita*^23^, demonstrated high identity on the nucleotide level of the identified ORFs with *A*. *aurita* genes (*aaqq1*, 92%; *aaqq2*, 97%; *aaqq3*, 86%) encoding for hypothetical proteins, which are assumed to be expressed in the planula, polyp, strobila, ephyra and medusa life stage. The presence of *aaqq1*, *aaqq2* and *aaqq3* in the *A*. *aurita* genome was further experimentally verified by PCR and subsequent sequence analysis using specific primers and genomic DNA isolated from two *A*. *aurita* life stages (lab-cultured polyp and ephyra) of native and germ-free (antibiotic (AB)-treated) animals (Fig. 2D). Additionally, reverse transcription (RT)-PCR analysis (two-step RT-PCR) demonstrated that all three ORFs are expressed in native and germ-free polyps and ephyrae. Moreover, first indications for different transcription patterns of the three ORFs in response to the respective life stages were obtained. Overall these results confirmed our assumption that the metagenomic ORFs are derived from the eukaryotic host and not from associated bacteria.

### QQ-ORF expression profiles under various stress conditions provide evidence for interkingdom signaling

**Figure 3:**
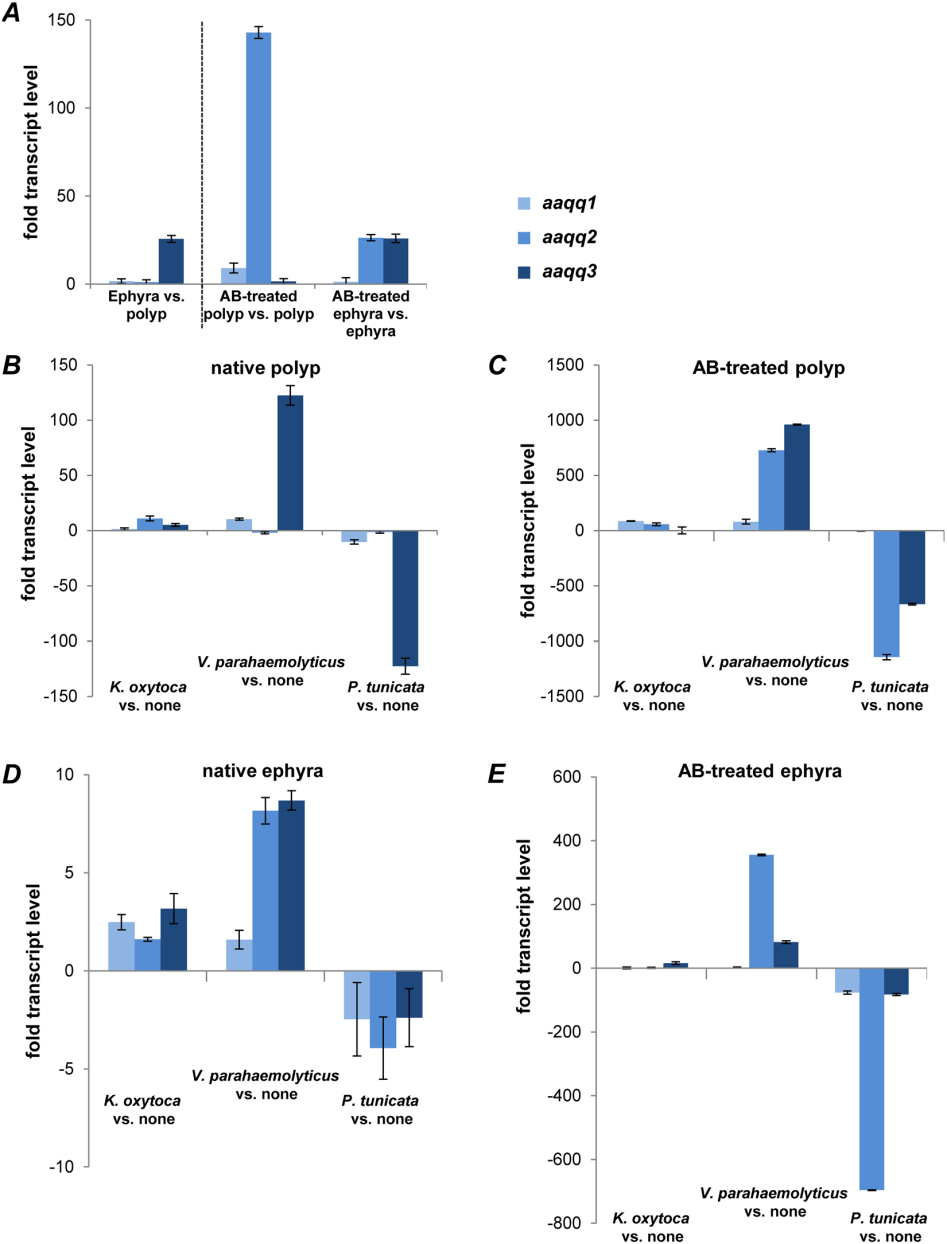
Relative transcript levels of QQ-ORFs in *A*. *aurita* polyps and ephyrae. Relative transcript levels of *aaqq1*, *aaqq2* and *aaqq3* were determined by qRT-PCR analysis in *A*. *aurita* polyps and ephyrae with at least three independent biological experiments, each with three technical replicates. (**A**) Polyps and ephyrae were kept under native and germ-free conditions. (**B–E**) Polyps and ephyrae were kept under native (**B+D**) and germ-free (**C+E**) conditions, additionally challenged with selected bacteria, *K*. *oxytoca*, *V*. *parahaemolyticus* and *P*. *tunicata*. Fold changes in transcript abundances were determined by comparison with the threshold cycle (Ct) of transcripts of the control gene *actin*.

We propose that different expression patterns of the identified QQ-ORFs are in part responsible for the different community structures of the microbiota (see^20^). Thus, we evaluated whether the identified QQ-ORFs are differentially expressed in the different life stages using quantitative (q)RT-PCR analyses with RNA isolated from native as well as germ-free polyps and ephyrae (Fig. 3A and Table S2). Comparing the relative expression of *aaqq1* and *aaqq2* in native polyps and ephyrae showed no drastic difference in transcript levels. In contrast, *aaqq3* showed 25-fold higher transcript levels in native ephyrae (Fig. 3A). Moreover, transcript levels of all QQ-ORFs were highly induced in germ-free animals compared to native animals indicating that the QQ-activities might be important as a defense mechanism, e.g. in the absence of the native microbiota. In detail, we observed a 140-fold increase in *aaqq2* transcripts in germ-free polyps, whereas a 25-fold increase in *aaqq2* and *aaqq3* transcripts was detected in germ-free ephyrae (Figs. 3A and S1). Though fitness-related traits were clearly unaffected in germ-free animals generated by antibiotic treatments (i.a. mortality frequency, growth rate, feeding rate), a stress response to this treatment cannot be completely excluded.

In addition, relative changes in transcript levels of QQ-ORFs were studied upon incubation of native and germ-free animals with *Klebsiella oxytoca* M5aI, *Vibrio parahaemolyticus* and *Pseudoalteromonas tunicata*. Recent studies on *A*. *aurita* bacterial colonization revealed that *K*. *oxytoca* has not been detected in any life stage of *A*. *aurita*^20^, and thus represents a foreign colonizer, which is generally able to form biofilms on different surfaces^24,25^. *V*. *parahaemolyticus*, an opportunistic pathogen^26^, has been isolated from an *A*. *aurita* polyp, and was exclusively detected on the polyp life stage^20^. *P*. *tunicata* was originally isolated from a medusa, but was also found in high abundance in ambient water as well as associated with all other life stages of *A*. *aurita*^20^, thus representing a commonly occurring bacterium. In native as well as germ-free polyps and ephyrae, incubation with the foreign bacterium *K*. *oxytoca* showed no drastic difference in transcript levels of all three ORFs (Figs. 3B–E and S1). In response to the commensal *P*. *tunicata*, transcription of QQ-ORFs in native and germ-free animals of both life stages was generally decreased compared to unchallenged animals (Fig. 3B–E). Native polyps even showed a 120-fold decrease in transcript levels of *aaqq3* (Fig. 3B). Germ-free polyps showed a similar response of *aaqq3* expression to *P*. *tunicata* as well as reduced *aaqq2* transcript levels (Fig. 3C). Such a response to *P*. *tunicata* was also observed for *aaqq2* in germ-free ephyrae (700 times reduction; Fig. 3E) indicating that expression of the QQ-ORFs is significantly down regulated in the presence of the commonly occurring bacterium. In contrast, challenging native as well as germ-free animals with the pathogenic *V*. *parahaemolyticus* caused significant induction of *aaqq2* and *aaqq3* transcription (Fig. 3B–E). In germ-free polyps, the response to *V*. *parahaemolyticus* was most drastic (Fig. 3C). Similar trends for differential gene expression of *aaqq2* and *aaqq3* were monitored in ephyrae (Fig. 3D, E).

In summary, challenging with foreign, commensal and opportunistic pathogenic colonizers resulted in different expression patterns of the three QQ-ORFs. In general, polyps showed a stronger response to the incubation with the three selected bacteria than the ephyrae stage. Moreover, it is remarkable that germ-free animals showed even more drastic changes in transcript levels compared to native animals (one order of magnitude higher) arguing for a protective effect of the microbiota on the host. In particular, the results in response to *V*. *parahaemolyticus* indicate that AAQQ2 and AAQQ3 might act as host defense proteins against (opportunistic) pathogens.

### Effects of immobilized QQ proteins on the native associated microbiota of *A. aurita*

To ultimately verify the effects of *A*. *auritas* QQ activities in shaping the associated microbiota, the actual impact of the identified AAQQ proteins on the composition of the associated microbial community of native *A*. *aurita* polyps was elucidated. The respective MBP-QQ fusion proteins and MBP as control protein were purified and immobilized on the surface of culture dishes by chemically cross-linking the protein to the pretreated surface, since the host is currently not genetically manipulable. In addition, a bacterial derived quorum quenching protein, QQ-2^27^, was immobilized as control. This metagenomic-derived QQ protein has recently been demonstrated to show highly efficient quenching activities against the signaling molecules AHL and AI-2; and thus resulted in highly effective inhibition of biofilm formation of various bacteria including pathogens^27^.

**Figure 4:**
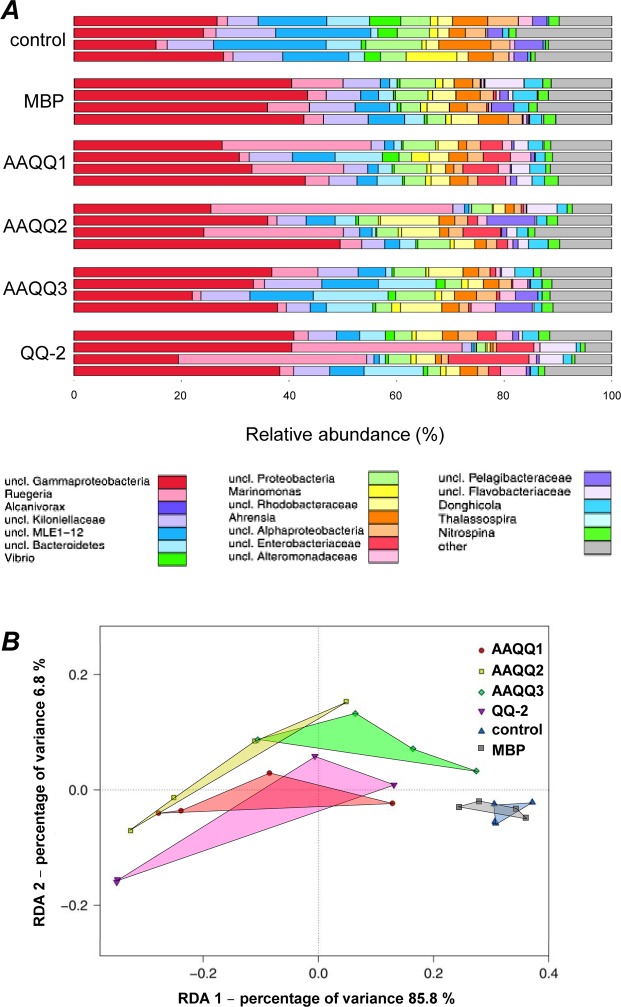
Composition of microbiota associated with *A*. *aurita* polyps after treatment with QQ-proteins. Microbial communities from native *A*. *aurita* polyps kept in presence of QQ-proteins AAQQ1, AAQQ2 and AAQQ3 as well as QQ-2 for 48 h were analyzed by sequencing the V1-V2 region of the 16 S bacterial rRNA gene. (**A**) OTU (97% pairwise similarity cut-off) abundances were summarized at genus level and normalized by the total number of reads per sample. Bar plots are grouped according to sample type each including 4 replicates. (**B**) Graphical representation (distance plots) of redundancy analysis (RDA) model of Hellinger-transformed OTU abundances. Each point represents the whole microbial community of an individual sample. Groups of related sample points are framed by polygons filled with the corresponding color.

After 48 h incubation in the presence of immobilized MBP-QQ proteins, the effect on the community structure was studied by amplicon sequencing of the V1-V2 region of the 16 S gene of the associated microbiota. After processing of sequence data, all samples had four analyzable replicates. Abundances of OTUs (97% sequence similarity) between individual samples have been determined and are depicted on genus level in Fig. 4A.

In general, at the genus level only slight differences in the diversity between untreated control polyps, MBP-treated and MBP-QQ-treated polyps were determined. However, the abundances of single genera were particularly affected due to the additional QQ activity (treatment), while differences between the different QQ proteins were marginal. Main representative genera in the microbiota of untreated and MBP-treated polyps were numerous unclassified γ-Proteobacteria, unclassified cyanobacterial MLE1–12, unclassified Bacteroidetes and *Marinomonas*. Main bacterial representatives of MBP-QQ-treated polyps were unclassified γ-Proteobacteria, *Ruegeria*, unclassified α-Proteobacteria, unclassified Enterobacteraceae, *Donghicola*, *Ahrensia* and *Nitrospina*. A more detailed differentiation between sample types was achieved by examining the beta diversity (distribution of individual OTUs across samples) explained by 86% of the variance in the data (Fig. 4B). Table S3 lists the results of corresponding pairwise factor level combinations. The data show that the presence of MBP-QQ proteins and their respective activity affected the microbiota of polyps, although those effects were considerably lower than in response to antibiotic treatment (Fig. S4). The variance in microbial patterns of QQ-treated polyps was higher than in control polyps.

Furthermore, indicator OTUs were identified in the microbial communities of control and QQ-treated polyps. The indicator analysis revealed specific community patterns linked to QQ treatment (Table S4). The presence of QQ proteins led to significant reduction of Cyanobacteria (representatives of classes 4C0d-2 and BD1–5) and α-Proteobacteria (e.g. *Ahrensia*) as well as *Vibrio*, *Bdellovibrio* and *Roseivirga*. Moreover, the presence of QQ proteins resulted in significant occurrence of Flavobacteriia, γ-Proteobacteria and α-Proteobacteria (mainly Rhodobacterales) (Table S4). Seven indicator OTUs were identified, which were absent in control polyps and exclusively appeared in major numbers after QQ treatment, however in different abundances (Table S5). In particular, α-Proteobacteria of the order Rhodobacterales are only present in QQ-treated polyps.

In conclusion, in response to immobilized recombinant MBP-QQ proteins, the polyp associated microbiota was restructured. Significant changes in abundance and OTU composition were observed in comparison with control polyps (native and MBP-treated), whereas only slight differences in microbial communities between the different QQ-treatments were observed. Consequently, the three externally added recombinant *A*. *aurita* QQ proteins significantly affected the community structure of polyp-associated microbiota clearly showing that QQ activities are in principle able to shape microbial communities on *A*. *aurita*.

## Discussion

Understanding the complex functional interactions between a host and its associated microbiota can provide important insights into symbiotic and pathogenic relationships, host immune response, development of host diseases, and might reveal so far unknown strategies for the development of novel therapeutics. The study of such complex interactions requires simple model organisms that allow the identification of key mechanisms and fundamental principles that possibly underlie all host-microbe interactions. *A*. *aurita* belongs to an important evolutionary old phylum that could potentially provide key insights into such interactions due to its morphological simplicity, but complex life cycle. Moreover, the moon jelly is constantly exposed to changing environmental conditions, and thus changing colonizer pools. During its life cycle and life span, *A*. *aurita* has to effectively distinguish between, and react to beneficial and harmful colonizers. Currently, there are no reports on *A*. *auritas* innate immune components. Consequently, we can only assume that highly conserved cell wall components of the bacteria (microbe/pathogen associated molecular patterns, MAMPs/PAMPs) are recognized by pattern recognition receptors (PRRs) of the host, ultimately leading to a response of the host due to facing bacteria. Based on the identification and demonstration of the effects of the three *A*. *aurita*-derived QQ-ORFs on the associated microbiota, we hypothesize that host response might also include expression of QQ-ORFs besides classical immune reactions^15,28^. Moreover, QQ might be a common fundamental strategy of eukaryotic hosts to shape their microbial community and help to maintain the healthy balance of the host by impacting the colonizers cell-cell communication. In the meantime, several examples have been reported, where the interkingdom communication can lead to specific adjustment and physiological adaptations of the host, e.g. AHLs are known to modulate immunity and development in plants (reviewed by^29^) or immune response and inflammatory signaling pathways of invertebrates (reviewed by^30,31^).

Here, we obtained several lines of evidence that the three identified host-derived QQ proteins shape the associated microbiota and are feasibly involved in pathogen defense. First, all three identified *A*. *aurita* QQ proteins were clearly demonstrated to interfere with both, the intra-species signaling molecule 3-oxo-C6 homoserine lactone (AHL of *V*. *fischeri*) of Gram-negative bacteria as well as with the universal signaling molecule AI-2. Effecting both signal molecules is not too surprising, since we recently identified various metagenomic-derived enzymes (i.e. oxidoreductases), which are able to modify both signaling molecules, and thus drastically interfered QS-based biofilm formation, although the biochemical mechanism of the interference is currently not known^27^. Downstream effects on biofilm formation have been also shown for the *A*. *aurita* derived QQ-proteins (Fig. S3). Thus, it is conceivable that QQ proteins can affect both signaling molecules leading to interfered bacterial communication important for host colonization on *A*. *aurita*. It is current knowledge that eukaryotes are able to produce QQ compounds, which mimic, modify or degrade signaling molecules. The probably best studied eukaryotic host using QQ is the red alga *Delisea pulchra*. The alga secretes brominated furanones, which mimic AHL molecules. The alga releases those brominated furanones, thus inhibiting multiple AHL-dependent processes of bacterial colonizers to protect from fouling microorganisms^17,32^. So far, there are only two eukaryotic QQ enzyme classes identified. Paraoxonases (PONs) have been identified in mammals, other vertebrates and invertebrates for being capable of hydrolyzing the homoserine lactone ring of AHLs^33,34^. PONs appear to be most active with long-chain AHL molecules, often typical for pathogens, e.g. *Pseudomonas aeruginosa* to regulate its virulence and biofilm formation^19^. Besides very recently, an oxidoreductase was identified in the fresh water polyp *Hydra vulgaris* transforming 3-hydroxy-homoserine lactone of its main colonizer *Curvibacter sp*. to 3-oxo-homoserine lactone. This modification of the *Curvibacter sp*. signaling molecule led to a phenotypic switch of the bacterium, ultimately resulting in reduced bacterial colonization of the host^18^. Unfortunately, we are currently not able to link the sequence information of the identified *A*. *aurita* QQ-ORFs to a predicted function; however we demonstrated that the QQ-proteins affect the natural microbiota. AAQQ1 shows about 30 similar hits in the compagen/Aurelia database, and thus appears to be relatively abundant in the *A*. *aurita* genome. Besides, there are homologous proteins in the genomes of the coral *Acropora* (XP_015752345) and the fresh water polyp *Hydra* (XP_012554234). AAQQ1 is predicted to be a part of a retrotransposon (jockey-like mobile element). Taking in addition the QS interfering activity in consideration, it is attractive to speculate that AAQQ1 does not longer function as mobile element in *A*. *aurita*, but the host has overtaken (a part of) the ORF for its own purpose as defense mechanism. AAQQ2, the SPYR domain-containing protein, which also might be involved in immune defense, is also similar to a protein in the fresh water polyp *Hydra* (XP_002159293). Without a published *A*. *aurita* genome, we are far away from predicting the natural function of the QQ proteins, however comparative sequence analysis indicates that QQ proteins might be a fundamental defense strategy against pathogens in basal metazoans.

Second, most of the *Aurelia*-derived identified QQ ORFs are differentially expressed in the benthic polyp and the pelagic ephyra arguing for a potential role of the QQ proteins for shaping the specific microbiota of the respective life stage. Comparing the expression profiles of the QQ-ORFs in both life stages, in particular the increased transcript levels of *aaqq3* in ephyrae indicated relevance for a potential function of AAQQ3 for microbial colonization of ephyrae, which might result in the life stage-specific bacterial colonization^20^ (Fig. S1). It is also conceivable that the QQ defense against bacteria is increased in ephyrae as an additional host defense mechanism in order to ensure that offspring are protected against pathogens, and develop to mature, reproductive medusa; ultimately ensuring survival of next generations. Considering the aspect of natural associated microbiota as a protective shield, by comparing untreated with germ-free animals, demonstrated highly increased expression levels of particularly *aaqq2* in germ-free polyps and *aaqq2/aaqq3* in germ-free ephyrae. Accordingly, the self-protection of the moon jelly against bacteria by QQ appears to be important in germ-free animals. We hypothesize that in general; the host-associated microbiota offers a protective effect against potential harmful colonizers and supports the host in maintaining a healthy homeostasis. Protective effects by epi- and endobiotic microbes against various potentially harmful microorganisms are well known for several eukaryotes, e.g. coral, seaweed and mouse^35,36^. For instance, the study by Schuijt *et al*. identified the intestinal microbiota as a protective mediator during pneumococcal pneumonia in mouse by enhancing alveolar immune response^36^. Third, the identified QQ-ORFs were differentially expressed in *A*. *aurita* polyps and ephyrae in response to incubation with selected bacteria, specifically the commensal *P*. *tunicata* and the potential pathogen *V*. *parahaemolyticus*. Incubation with the commensal *P*. *tunicata* led to reduced transcript levels of all QQ-ORFs, implying that constant presence of a non-pathogenic bacterium results in a decreased expression of the QQ-ORFs indicating that the animal does not defend against commensal colonizers. In contrast, challenging with the potential pathogen *V*. *parahaemolyticus* led to a strong response, high up-regulation of especially *aaqq2* and *aaqq3* transcript levels (Fig. S1). Apparently, *V*. *parahaemolyticus* is recognized by the animal as potentially harmful and *A*. *aurita* activates its QQ repertoire to repel the invader in addition to the immune system.

Fourth, experiments with *A*. *aurita* polyps in the presence of immobilized recombinant QQ proteins demonstrated that the polyp-associated microbiota is modulated in response to the present QQ activity. It is notable that Cyanobacteria, several unclassified α-Proteobacteria, *Vibrio*, *Bdellovibrio* and *Roseivirga* significantly decreased in their abundance after QQ treatment, whereas Flavobacteria, various γ-Proteobacteria and α-Proteobacteria, i.a. *Ruegeria*, *Donghicola* and *Roseovarius* increased in their abundances. Almost all of those representatives encode QS genes to communicate in the bacterial community using AHL or/and AI-2^37–41^. Thus, it is likely that the host QQ proteins indeed interfere with the respective signaling molecules synthesized by the bacterial colonizers, and thus affect colonization of the host. Besides, several indicator species (OTUs) linked to the QQ treatment are known to be symbionts of marine eukaryotes^42–44^. For instance, tropodithietic acid (TDA)-producing *Ruegeria* strains of the Roseobacter clade have primarily been isolated from marine aquaculture and are able to switch between a “swim-or-stick” mode when colonizing a host surface. Moreover, these strains have probiotic potential due to inhibition of fish pathogens, ultimately contributing to host fitness^45^. Though, we are fully aware of probably using unnatural concentrations of QQ proteins in the experiment (maximal 3 nmol protein/cm^2^) and thus not disclosing the real dimension of the effects of the QQ protein activities. However, we expect that the artificial sea water is not representing the optimal conditions for the respective enzyme activities. Furthermore, the 48 h incubation potentially significantly reduces enzyme activities. Besides, immobilization of proteins in seawater does not allow Co-factor regeneration as required, e.g. for control protein QQ-2, which requires NADH for enzyme activity. Overall, our data illustrate that the impact of additional QQ proteins showing highest expression levels in quantitative analysis (see Fig. S1), and thus might represent important QQ-ORFs in native animals, indeed resulted in restructuring of the bacterial community compared to the control polyps (native polyps and in presence of immobilized control protein MBP). Consequently, we can only speculate on the effect of the community restructuring on the fitness of *A*. *aurita* due to the QQ treatment.

In conclusion, based on all findings of the present study we hypothesize that QQ is an additional host mechanism in *A*. *aurita* to respond to surrounding bacteria, and defend specific bacteria to ultimately maintain a healthy microbiota. The identified QQ-ORFs are differentially expressed during the life cycle of *A*. *aurita*, when the animal is facing different challenging conditions, to modulate its microbiota. Moreover, the differential expression of those ORFs appears to be the result of the recognition of specific cell characteristics of confronting bacteria leading to bacterial attraction or defense. Here, to the best of our knowledge, we showed for the first time that the bacterial community structure changed upon external QQ activity derived from the host. Consequently, the present observations provide insights into complex interkingdom interactions of the host *A*. *aurita* with its residing microbiota. The present study and the recent study of Pietschke *et al*.^18^ on *Curvibacter spec*. and hydra show that in metaorganisms, bacterial communication and its host derived modulation might be a strategy of host-microbe communication and possibly evolved early during metazoan evolution as a fundamental mechanism in host–microbe interactions. Consequently, comparative studies with other basal metazoans are currently underway and might provide more information on QQ as a fundamental defense strategy.

## Materials and Methods

Bacterial strains and plasmids are listed in Table S6.

### Bacterial growth conditions

The media used in this study were Luria-Bertani (LB) medium^46^ and Marine Bouillon (MB) (Roth, Karlsruhe, Germany). When indicated, the LB medium was supplemented with final concentrations of the following antibiotics: ampicillin 100 µg/mL, kanamycin 30 µg/mL or chloramphenicol 12.5 µg/mL.

Challenging experiments were performed with *K*. *oxytoca* M5aI, *V*. *parahaemolyticus* and *P*. *tunicata*, grown in 20 mL MB medium, except *K*. *oxytoca* in LB medium, at 30° and 120 rpm overnight. *A*. *aurita* polyps were incubated 30 min with 10^8^ cells/mL of the respective strain at 20 °C in 3 mL artificial sea water, washed twice with sterile artificial seawater to remove bacteria and used for isolation of mRNA as described below to analyze changes in transcript levels of QQ-ORFs.

### Sampling and enrichment from *A. aurita*

Individual *A*. *aurita* (Linnaeus) medusae (mean size of umbrella diameter 26 cm) were sampled from one location in the Kiel Bight, Baltic Sea (54°19.4´N, 10°08.5´E) in May 2009 using a dip net. The animals were transported immediately to the laboratory, washed thoroughly with sterile-filtered artificial seawater to remove non-associated microbes. Mucus from ex- and subumbrella was prepared by scraping and sampling the slime. Mucus was used for DNA isolation as well as enrichment of bacteria on MB agar plates. Additionally, enrichment of bacteria was performed with polyps. Pure cultures were analyzed by 16 S rDNA and taxonomically classified *via* BLAST search using the NCBI 16 S rRNA database. The isolate from the medusa was classified as *Pseudoalteromonas tunicata* (Accession No. JX287299), the bacterial isolate from the polyp was classified as *Vibrio parahaemolyticus* (Accession No. JX287307)^20^.

### *A*. *aurita* polyp husbandry and generation of germ-free polyps

Husbandry and generation of germ-free polyps is described in detail in Weiland-Bräuer *et al*.^20^. The absence of bacteria was confirmed by plating homogenized animals on R2A agar plates (Roth, Karlsruhe/Germany). After incubation at 19 °C for 5 days, the colony forming units (CFU) were determined. Absence of CFUs indicated successful antibiotic treatment. Moreover, the full length bacterial 16 S rRNA gene was amplified using GoTaq Polymerase (Promega, Madison, USA) and Bacteria-specific primer 27 F (5′-AGAGTTTGATCCTGGCTCAG-3′) and the universal primer 1492 R (5′-GGTTACCTTGTTACGACTT-3′). Samples without successful amplification were graded as germ-free. Additionally, for culture-independent analysis, total DNA was extracted and the hypervariable V1-V2 region of the 16 S rRNA gene amplified as described below.

### Nucleic acid isolation

DNA of *A*. *aurita* mucus (1 mL v/v) and polyps (10 polyps for each preparation) was isolated using Wizard Genomic DNA Purification Kit (Promega, Madison, USA). mRNA of 20 polyps was isolated using the illustra™ QuickPrep Micro mRNA Purification Kit (GE Healthcare, Chalfont St. Giles, UK). Fosmid DNA was isolated from 5 mL overnight cultures using High-Speed-Plasmid-Mini Kit (Avegene, Taipei, Taiwan).

### PCR amplification

QQ-ORFs *aaqq1*, *aaqq2* and *aaqq3* were amplified by PCR with primers listed in Table S7 from 5 ng genomic DNA isolated from *A*. *aurita* medusa, polyps and ephyrae. For RT-PCR, 50 ng cDNA of polyps and ephyrae were used. cDNA was synthesized with Fermentas First Strand cDNA Synthesis Kit using random hexamer primers after DNase I treatment with addition of RiboLock™ RNase Inhibitor (Thermo Fisher Scientific, Darmstadt, Germany). Before use of cDNA in quantitative PCR, samples were purified using NucleoSpin Gel and PCR Clean-up kit (Macherey-Nagel, Düren, Germany). qRT-PCRs were performed with three independent RNA preparations each with three technical replicates on an Applied Biosystems 7300 Real-Time PCR System using cDNA prepared with PlatinumR SYBRR Green qPCR SuperMix-UDG with ROX (Thermo Fisher Scientific, Darmstadt, Germany). The fold change in transcript abundance for QQ-ORFs was determined by comparison with the threshold cycle (C_t_) of transcripts of the control housekeeping gene β-actin. The fold change in the abundance of a transcript was calculated using the formula *fold change* = *2*^*−ΔΔCt*^ as described^47,48^.

### Construction of metagenomic large insert library

A large-insert fosmid library was constructed using Copy Control^TM^ Fosmid Library Production Kit with vector pCC1FOS (Epicentre, Madison, USA) with modifications as mentioned in Weiland-Bräuer *et al*.^49^. The insert-flanking T7 promoter of the vector pCC1FOS allows transcription of the cloned foreign genes in an *E*. *coli* background, since the interaction of the *E*. *coli* RNA polymerase with the T7 promoter is known for a long time^50,51^.

### Quorum quenching assay

QQ assays using strains AI1-QQ.1 and AI2-QQ.1 were performed with cell-free supernatants and cell extracts of metagenomic clones and purified proteins as previously described in Weiland-Bräuer *et al*.^21^. In addition, as a control purified QQ proteins were tested with our control strain XL1-Blue/pZErO-2 to exclude possible effects on the toxicity of the lethal protein (e.g., by degradation or transportation out of the cell and thus interfering with the screening approach)^21^.

### Molecular characterization of quorum sensing interfering clones

In order to identify the respective ORFs of fosmids conferring QQ activity, subcloning and subsequent sequencing was used as previously described in Weiland-Bräuer *et al*.^27^.

### Expression and purification of QQ proteins as Maltose Binding Protein (MBP)-fusions

Putative QQ-ORFs were PCR-cloned into pMAL-c2X N-terminally fusing the QQ-ORFs to MPB using ORF-specific primers adding restriction recognition sites flanking the ORFs (see Table S6); overexpressed and purified as recently described in Weiland-Bräuer *et al*.^27^.

### Covalent immobilization of QQ proteins

Polystyrene Petri dishes 35 × 10 mm (Greiner, Kremsmünster, Austria) were coated by the company Surflay Nanotec (Berlin,Germany) with ethyleneiminepolymers (PEI) according to the previously published Layer-by-Layer method^52^. Immobilization of QQ proteins with glutaraldehyde (5% v/v) was performed as described previously^27^. Polyps (10 polyps per dish, 6 dishes) were incubated in artificial seawater for 48 h in presence of QQ proteins prior to microbial DNA isolation for HTP sequencing analysis.

### HTP sequencing analysis

Fosmid pCC1FOS.2 was isolated as mentioned above and provided for *de novo* sequencing on 454 GS-FLX-Titanium Sequencer (454 Life Sciences, Roche) according to the manufacturer. Bacterial DNA was isolated as described above and used for generation of PCR amplicon libraries using primers V1_A_Pyro_27F (5′-CGTATCGCCTCCCTCGCGCCATCAGTCAGAGTTTGATCCTGGCTCAG-3′) and V2_B_Pyro_27F (5′- CTATGCGCCTTGCCAGCCCGCTCAGTCAGAGTTTGATCCTGGCTC AG-3′). Amplicons were size checked and purified using MinElute Gel Extraction Kit (Qiagen). Purified amplicon DNAs were quantified using Quant-iT PicoGreen kit (Invitrogen) and equal amounts of purified PCR products were pooled for subsequent pyrosequencing. Pyrosequencing using the 454/Roche GS FLX chemistry was carried out according to the manufacturer’s instructions using the GS FLX Titanium series kit in the 454 GS-FLX-Titanium Sequencer (454 Life Sciences, Roche) at Max-Planck Institute for Evolutionary Biology in cooperation with Dr. S. Künzel.

### Data processing and bioinformatics

All steps of sequence processing were conducted with the program mothur v1.27.0^53^. The Greengenes reference taxonomy^54^ available on http://www.mothur.org/wiki/Greengenes-formatted_databases was used with reference sequences trimmed to the V1-V2 primer region to improve accuracy of classification^55^. A random subset of 1,351 sequences per sample (corresponding to the smallest number of reads across samples) was generated to eliminate bias due to unequal sampling effort (sub.sample). The aligned and subsampled dataset was used to compute a distance matrix (dist.seqs) for binning sequences into operational taxonomic units (OTU) by average neighbor clustering (cluster.split). 662 OTUs at a 97% similarity threshold (roughly corresponding to species level) distinction were considered. All downstream computations were performed in R v2.15.1^56^ as described in^20^. Indicator OTU analysis was also performed in R. Mothur shared OTU tables were imported into R using the phyloseq package^57^. Subsequently, the multipatt function of the indicspecies package^58^ was used to identify OTUs that were significantly associated with the replicate groups.

Sequence data were deposited in the NCBI Sequence Read Archive (accession number SRP120156 or bioproject number PRJNA414615).

### Data deposition

The sequences reported in the paper have been deposited in the GenBank database (Accession Nos. JX274296, JX274297, JX274298). Full length sequence of fosmid 2 insert is provided as supplemental dataset.

## Supplementary information

Supplemental information

Dataset 1

## Data Availability

All data generated or analyzed during this study are included in this published article (and its supplementary information files).
