# Supplementary material for: Potential role of host-derived quorum quenching in modulating bacterial colonization in the moon jellyfish Aurelia aurita
Source: Sci Rep. 2019 Jan 10;9:34. doi: 10.1038/s41598-018-37321-z (PMC6328592; doi:10.1038/s41598-018-37321-z)
Supplement: Supplementary file 1 — Supplemental information [file 41598_2018_37321_MOESM1_ESM.docx]

# Potential role of host-derived quorum quenching in modulating bacterial colonization in the moon jellyfish *Aurelia aurita*

Nancy Weiland-Bräuer, Martin A. Fischer, Nicole Pinnow, and Ruth A. Schmitz*

**Supplemental information**

**Fig. S1: Differential expression of QQ-ORFs in *A. aurita*.** The evolutionary ancient moon jellyfish *A. aurita* shows a complex life cycle with an alteration of benthic polyps and pelagic medusae, which develop from ephyrae (photographs and drawing have been created by contributing author Weiland-Bräuer). Heat maps summarize the differential expression of QQ-ORFs *aaqq1*, *aaqq2* and *aaqq3* in the life stages polyp and ephyra under native and AB-treated conditions.

**Fig. S2: Construction of QQ-fusion proteins.** In expression vector pMAL-c2X, QQ-ORFs were cloned between the genes *malE* (Maltose Binding protein, MBP) and *lacZα* (β-galactosidase subunit α) to N-terminally fuse the QQ proteins to MBP. Relevant sequence information are presented for each QQ-ORF.

**Fig. S3: Inhibition of biofilm formation by MBP-AAQQ proteins.** (*A*) Biofilm-forming strains (*Pseudomonas aeruginosa, Bacillus subtilis* and Staphylococcus *aureus*) were grown in 96 well plates in minimal medium (*B. subtilis,* AB medium*;* *S. aureus* and *P. aeruginosa,* Caso bouillon medium) for 24 h and 80 rpm at 30 °C. Besides growth controls of the respective strains incubated without a supplement, purified MBP-AAQQ proteins were added to the cultures in amounts of 10 µg (■), 50 µg (■) and 100 µg (■). After 24 h incubation, the established biofilms were stained with crystal violet, subsequently solved in 96 % ethanol and absorbance of the solutions was measured at 590 nm as described in Weiland-Bräuer *et al*., 2016^1^. (*B)* Biofilm formation of *Klebsiella oxytoca* M5a1 expressing the AAQQ-ORFs from pMALc2X-fusion vectors was monitored after 48 h using Live/Dead viability Kit and Leica TCP Confocal Laser Scanning Microscope (Leica) and biofilm volume calculated using IMARIS software^1^. Scale bars represent 50 μm.

**Fig. S4: Redundancy analysis (RDA) model of Hellinger-transformed OTU abundances.** (*A*) Graphical representation (distance plots) of redundancy analysis (RDA) model of Hellinger-transformed OTU abundances. Each point represents the whole microbial community of an individual sample associated with polyps kept under native conditions in presence of immobilized QQ proteins; and induced sterile conditions. Groups of related sample points are framed by polygons filled with a corresponding color to elucidate distribution and variability of sample groups in the ordination space. (*B*) Results of pairwise tests in beta diversity analysis. Tests were conducted for the pairs of factor level combinations stated in column “Comparison”. Results at the 5 %-level for P-value and above 2 for F-value are significant.

**Tab. S1: Predicted 3D structures of *A. aurita* QQ proteins.** 3D structures were predicted using Phyre2 (http://www.sbg.bio.ic.ac.uk/phyre2/html/page.cgi?id=index) as shown in Fig. 2 based on protein models in the respective database. Information on respective template models is listed in the table.

**Table S2: Relative transcript levels of QQ-ORFs in polyps compared with ephyrae.** Relative transcript levels of QQ-ORFs in native and antibiotic (AB)-treated *A. aurita* polyps as well as ephyrae were determined by quantitative PCR analysis.

**Tab. S3: Results of pairwise tests in beta diversity analysis.** Tests were conducted for specified comparisons. Results significant at the 5 %-level for P-value and above factor 2 for F-value are in bold face type.

**Tab. S4: Indicator OTUs.** Significant indicator OTUs are listed for each single sample type and the QQ treatments.

**Tab. S5: Negative indicator OTUs.** Significant indicator OTUs are listed, which are absent in control polyps.

**Table S6: Bacterial strains and plasmids used in this study.**

**Table S7: Primers used in this study.**


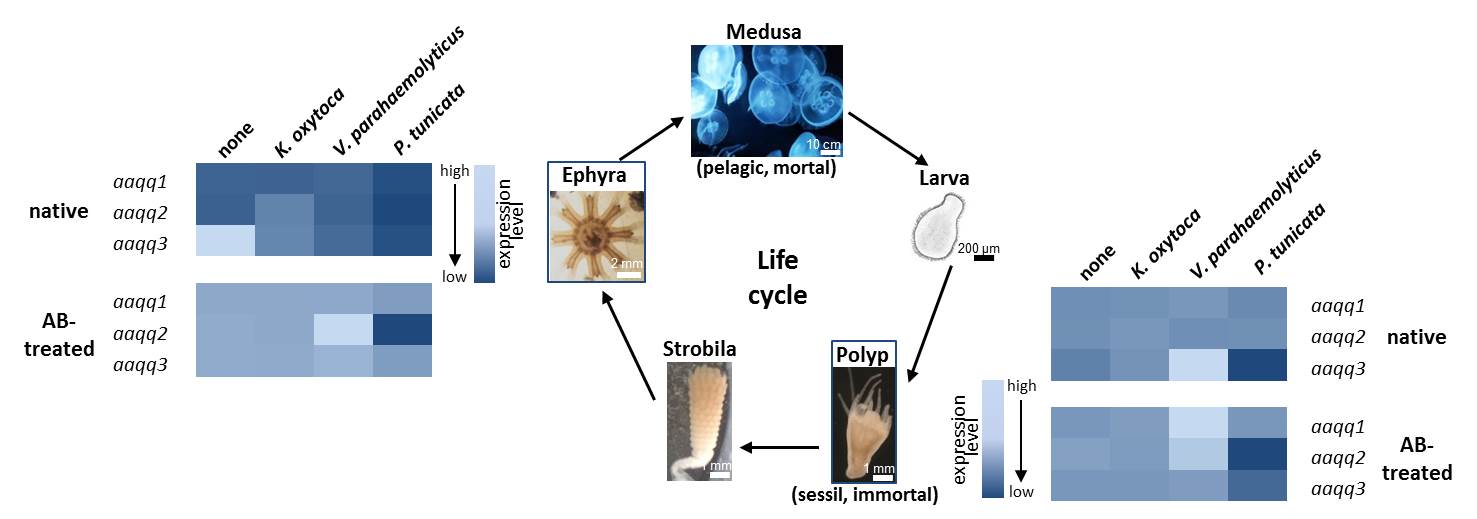


**Fig. S1: Differential expression of QQ-ORFs in *A. aurita*.** The evolutionary ancient moon jellyfish *A. aurita* shows a complex life cycle with an alteration of benthic polyps and pelagic medusae, which develop from ephyrae (photographs and drawing have been created by first author Weiland-Bräuer). Heat maps summarize the differential expression of QQ-ORFs *aaqq1*, *aaqq2* and *aaqq3* in the life stages polyp and ephyra under native and AB-treated conditions in response to the different challenge bacteria.


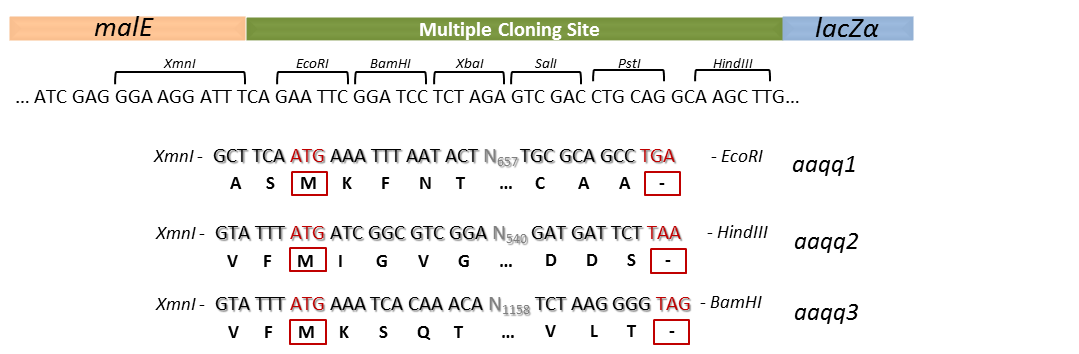


**Fig. S2: Construction of QQ-fusion proteins.** In expression vector pMAL-c2X, QQ-ORFs were cloned between the genes *malE* (Maltose Binding protein, MBP) and *lacZα* (β-galactosidase subunit α) to N-terminally fuse the QQ proteins to MBP. Relevant sequence information are presented for each QQ-ORF.


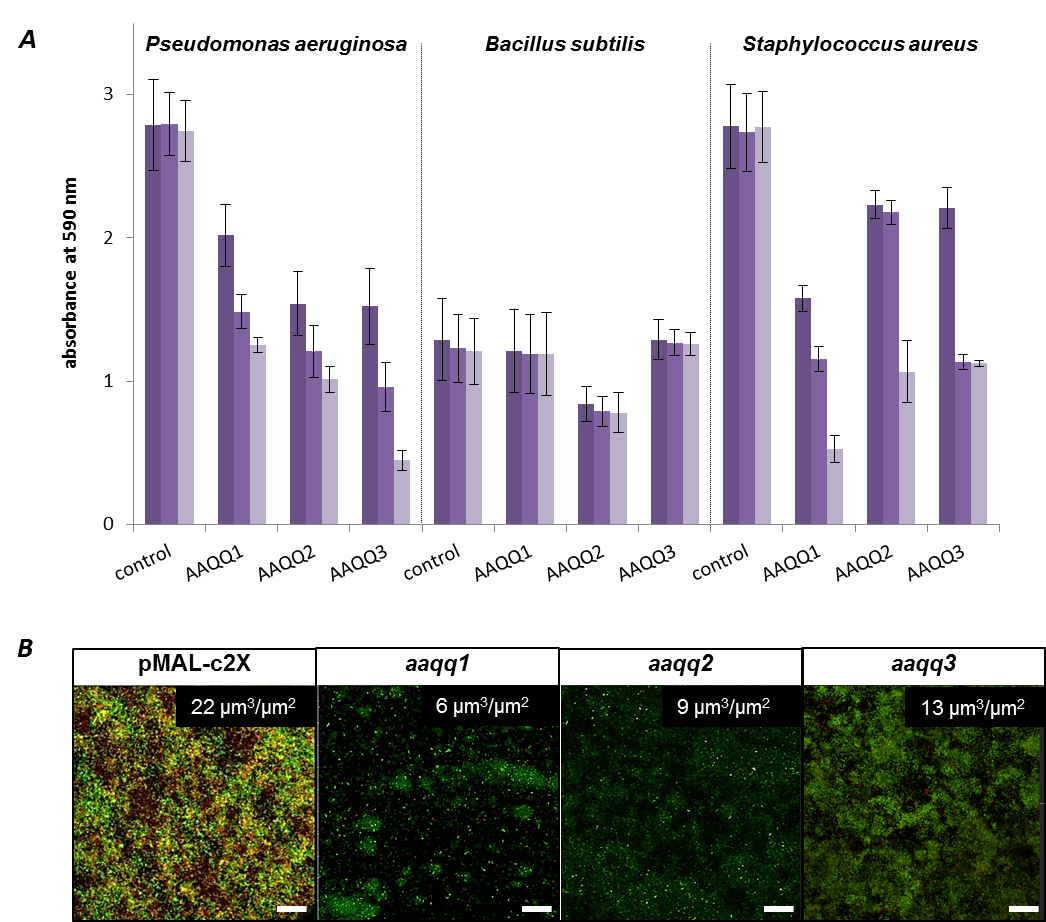


**Fig. S3: Inhibition of biofilm formation by MBP-AAQQ proteins.** (***A***) Biofilm-forming strains (*Pseudomonas aeruginosa, Bacillus subtilis* and *Staphylococcus aureus*) were grown in 96 well plates in minimal medium (*B. subtilis,* AB medium*;* *S. aureus* and *P. aeruginosa,* Caso bouillon medium) for 24 h and 80 rpm at 30 °C. Besides growth controls of the respective strains incubated without a supplement, purified MBP-AAQQ proteins were added to the cultures in amounts of 10 µg (■), 50 µg (■) and 100 µg (■). After 24 h incubation, the established biofilms were stained with crystal violet, subsequently solved in 96 % ethanol and absorbance of the solutions was measured at 590 nm as described in Weiland-Bräuer *et al*., 2016^1^. (***B)*** Biofilm formation of *Klebsiella oxytoca* M5a1 expressing the AAQQ-ORFs from pMALc2X-fusion vectors was monitored after 48 h using Live/Dead viability Kit and Leica TCP Confocal Laser Scanning Microscope (Leica) and biofilm volume calculated using IMARIS software ^1^. Scale bars represent 50 μm.

^1^ Weiland-Bräuer, N., Kisch, M. J., Pinnow, N., Liese, A., & Schmitz, R. A. (2016). Highly effective inhibition of biofilm formation by the first metagenome-derived AI-2 quenching enzyme. *Frontiers in microbiology*, *7*, 1098.


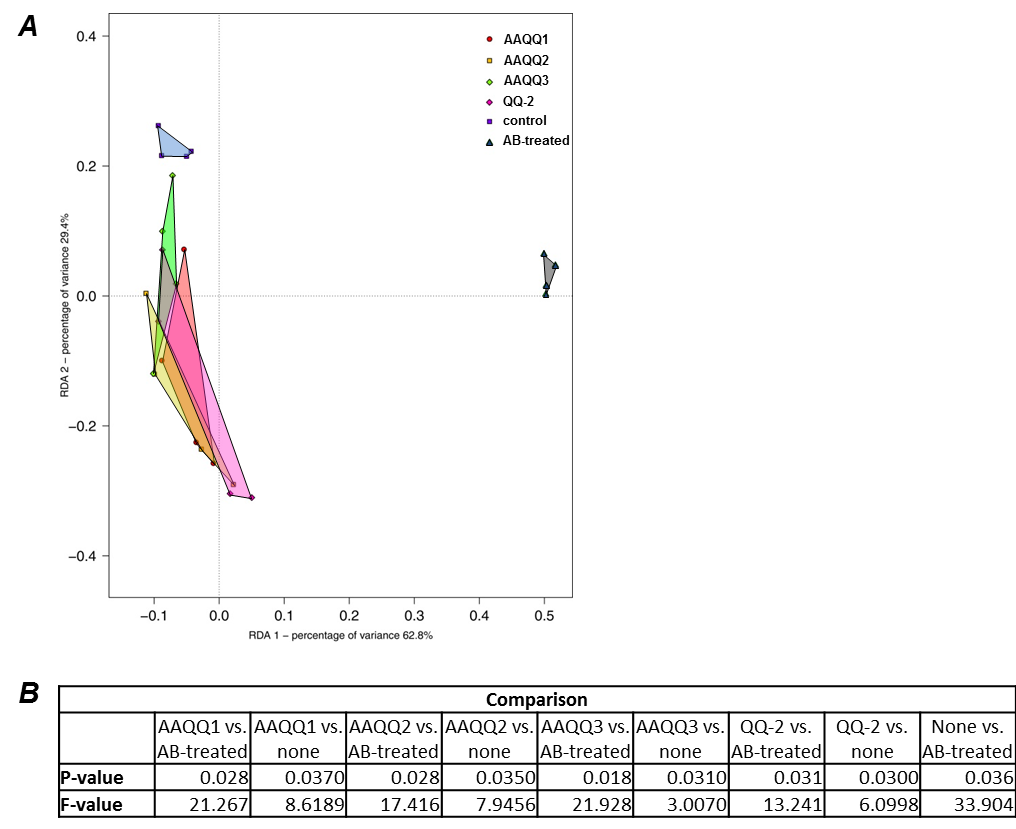


**Fig. S4: Redundancy analysis (RDA) model of Hellinger-transformed OTU abundances. (*A*)** Graphical representation (distance plots) of redundancy analysis (RDA) model of Hellinger-transformed OTU abundances. Each point represents the whole microbial community of an individual sample associated with polyps kept under native conditions in presence of immobilized QQ proteins; and induced sterile conditions. Groups of related sample points are framed by polygons filled with a corresponding color to elucidate distribution and variability of sample groups in the ordination space. (***B***) Results of pairwise tests in beta diversity analysis. Tests were conducted for the pairs of factor level combinations stated in column “Comparison”. Results at the 5 %-level for P-value and above 2 for F-value are significant.

**Tab. S1: Predicted 3D structures of *A. aurita* QQ proteins.** 3D structures were predicted using Phyre^2^ (<http://www.sbg.bio.ic.ac.uk/phyre2/html/page.cgi?id=index>) as shown in Fig. 2 based on protein models in the respective database. Information on respective template models is listed in the table.

| **QQ protein** | **Model template** | **Characteristics of template model** | **Empirical factors** | |
| --- | --- | --- | --- | --- |
|  |  |  | **Confidence (%)** | **Coverage (%)** |
| **AAQQ1** | c6ar3A | - RNA binding protein - thermostable group II intron - reverse transcriptase | 96.5 | 43 |
| **AAQQ2** | d2fnja1 | - - Concanavalin A-like lectins /glucanases - SPRY domain | 100 | 64 |
| **AAQQ3** | c2gj2A | - metal binding protein - linked to white spot syndrome virus | 1.4 | 3 |

**Table S2: Relative transcript levels of QQ-ORFs in polyps compared with ephyrae.** Relative transcript levels of QQ-ORFs in native and antibiotic (AB)-treated *A. aurita* polyps as well as ephyrae were determined by quantitative PCR analysis.

| ***Aurelia aurita* polyps compared to ephyrae** | | |
| --- | --- | --- |
| **QQ-ORF** | **compared condition** | **fold regulation** |
| ***aaqq1*** | ephyra vs. polyp | 1.68 ± 1.28 |
|  | polyp vs. AB-treated polyp | 9.07 ± 2.79 |
|  | ephyra vs. AB-treated ephyra | 1.28 ± 2.26 |
| ***aaqq2*** | ephyra vs. polyp | 1.08 ± 1.34 |
|  | polyp vs. AB-treated polyp | 142.86 ± 3.34 |
|  | ephyra vs. AB-treated ephyra | 26.32 ± 1.75 |
| ***aaqq3*** | ephyra vs. polyp | 25.64 ± 1.93 |
|  | polyp vs. AB-treated polyp | 1.51 ± 1.61 |
|  | ephyra vs. AB-treated ephyra | 25.92 ± 2.44 |
| ***Aurelia aurita* untreated polyps incubated with selected bacteria** | | |
| **QQ-ORF** | **selected bacterial species compared with control** | **fold regulation** |
| ***aaqq1*** | incubated with *Klebsiella oxytoca* M5aI | 1.43 ± 0.99 |
|  | incubated with *Vibrio parahaemolyticus* | 10.37 ± 1.01 |
|  | incubated with *Pseudoalteromonas sp.* | - 10.35 ± 1.94 |
| ***aaqq2*** | incubated with *Klebsiella oxytoca* M5aI | 10.96 ± 2.21 |
|  | incubated with *Vibrio parahaemolyticus* | - 1.87 ± 1.08 |
|  | incubated with *Pseudoalteromonas sp.* | - 1.27 ± 0.98 |
| ***aaqq3*** | incubated with *Klebsiella oxytoca* M5aI | 5.10 ± 1.24 |
|  | incubated with *Vibrio parahaemolyticus* | 122.53 ± 8.89 |
|  | incubated with *Pseudoalteromonas sp.* | - 122.35 ± 7.25 |
| ***Aurelia aurita* AB-treated polyps incubated with selected bacteria** | | |
| ***aaqq1*** | incubated with *Klebsiella oxytoca* M5aI | 86.34 ± 1.18 |
|  | incubated with *Vibrio parahaemolyticus* | 960.74 ± 21.22 |
|  | incubated with *Pseudoalteromonas sp.* | - 2.88 ± 2.72 |
| ***aaqq2*** | incubated with *Klebsiella oxytoca* M5aI | 56.81 ± 12.81 |
|  | incubated with *Vibrio parahaemolyticus* | 727.59 ± 13.02 |
|  | incubated with *Pseudoalteromonas sp.* | - 1144.16 ± 23.34 |
| ***aaqq3*** | incubated with *Klebsiella oxytoca* M5aI | 1.89 ± 0.65 |
|  | incubated with *Vibrio parahaemolyticus* | 81.01 ± 4.01 |
|  | incubated with *Pseudoalteromonas sp.* | - 666.67 ± 7.53 |
| ***Aurelia aurita* untreated ephyrae incubated with selected bacteria** | | |
| ***aaqq1*** | incubated with *Klebsiella oxytoca* M5aI | 1.59 ± 0.39 |
|  | incubated with *Vibrio parahaemolyticus* | 2.48 ± 0.48 |
|  | incubated with *Pseudoalteromonas sp.* | - 2.47± 1.87 |
| ***aaqq2*** | incubated with *Klebsiella oxytoca* M5aI | 8.16 ± 0.10 |
|  | incubated with *Vibrio parahaemolyticus* | 1.61 ± 0.67 |
|  | incubated with *Pseudoalteromonas sp.* | -3.94 ± 1.59 |
| ***aaqq3*** | incubated with *Klebsiella oxytoca* M5aI | 8.69 ± 0.77 |
|  | incubated with *Vibrio parahaemolyticus* | 3.17 ± 0.50 |
|  | incubated with *Pseudoalteromonas sp.* | - 2.39 ± 1.48 |
| ***Aurelia aurita* AB-treated ephyrae incubated with selected bacteria** | | |
| ***aaqq1*** | incubated with *Klebsiella oxytoca* M5aI | 1.03 ± 2.94 |
|  | incubated with *Vibrio parahaemolyticus* | 2.11 ± 1.42 |
|  | incubated with *Pseudoalteromonas sp.* | - 76.37 ± 4.76 |
| ***aaqq2*** | incubated with *Klebsiella oxytoca* M5aI | 1.36 ± 0.74 |
|  | incubated with *Vibrio parahaemolyticus* | 355.57 ± 2.72 |
|  | incubated with *Pseudoalteromonas sp.* | - 696.41 ± 1.26 |
| ***aaqq3*** | incubated with *Klebsiella oxytoca* M5aI | 16.13 ± 4.13 |
|  | incubated with *Vibrio parahaemolyticus* | 81.69 ± 4.04 |
|  | incubated with *Pseudoalteromonas sp.* | - 82.25 ± 3.29 |

**Tab. S3: Results of pairwise tests in beta diversity analysis.** Tests were conducted for specified comparisons. Results significant at the 5 %-level for P-value and above factor 2 for F-value are in bold face type.

| **Comparison** | **F-value** | **P-value** |
| --- | --- | --- |
| control - AAQQ1 | **8.6189** | **0.0370** |
| control - AAQQ2 | **7.9456** | **0.0350** |
| control - AAQQ3 | **3.0070** | **0.0310** |
| control - QQ-2 | **6.0998** | **0.0300** |
| AAQQ1 - AAQQ2 | 0.5385 | 0.6160 |
| AAQQ1 - AAQQ3 | 1.9680 | 0.1770 |
| AAQQ1 - QQ-2 | 0.2072 | 0.9020 |
| AAQQ2 - AAQQ3 | 2.2676 | 0.1310 |
| AAQQ2 - QQ-2 | 0.4455 | 0.6250 |
| AAQQ3 - QQ-2 | 1.8262 | 0.1830 |

**Tab. S4: Indicator OTUs.** Significant indicator OTUs are listed for each single sample type and the QQ treatments.

| **Sample type** | **Indicator OTU** | **P-value** | **Taxonomy** | | | |
| --- | --- | --- | --- | --- | --- | --- |
|  |  |  | **Phylum** | **Class** | **Order** | **Genus** |
| **Control** | OTU00008 | 0.015 | Cyanobacteria | 4C0d-2 | MLE1-12 | unclassified |
|  | OTU00011 | 0.022 | Proteobacteria | Alphaproteobacteria | Rhizobiales | *Ahrensia* |
|  | OTU00019 | 0.047 | Proteobacteria | Gammaproteobacteria | Vibrionales | *Vibrio* |
|  | OTU00021 | 0.006 | Proteobacteria | unclassified | unclassified | unclassified |
|  | OTU00033 | 0.005 | Proteobacteria | unclassified | unclassified | unclassified |
|  | OTU00034 | 0.019 | Proteobacteria | Gammaproteobacteria | Oceanospirillales | unclassified |
|  | OTU00036 | 0.015 | Proteobacteria | Alphaproteobacteria | unclassified | unclassified |
|  | OTU00040 | 0.006 | Bacteroidetes | Cytophagia | Cytophagales | *Roseivirga* |
|  | OTU00046 | 0.024 | Proteobacteria | Alphaproteobacteria | unclassified | unclassified |
|  | OTU00049 | 0.005 | GN02 | BD1-5 | unclassified | unclassified |
|  | OTU00059 | 0.005 | Proteobacteria | Alphaproteobacteria | unclassified | unclassified |
|  | OTU00060 | 0.005 | Proteobacteria | unclassified | unclassified | unclassified |
|  | OTU00109 | 0.044 | Proteobacteria | Alphaproteobacteria | Kiloniellales | unclassified |
|  | OTU00151 | 0.007 | Cyanobacteria | 4C0d-2 | MLE1-12 | unclassified |
|  | OTU00154 | 0.022 | Cyanobacteria | 4C0d-2 | MLE1-12 | unclassified |
|  | OTU00157 | 0.006 | Proteobacteria | Deltaproteobacteria | unclassified | unclassified |
|  | OTU00187 | 0.013 | Proteobacteria | Alphaproteobacteria | BD7-3 | unclassified |
|  | OTU00228 | 0.003 | Proteobacteria | unclassified | unclassified | unclassified |
|  | OTU00263 | 0.006 | Proteobacteria | Alphaproteobacteria | unclassified | unclassified |
|  | OTU00266 | 0.019 | Proteobacteria | Deltaproteobacteria | Bdellovibrionales | *Bdellovibrio* |
|  | OTU00279 | 0.007 | Cyanobacteria | 4C0d-2 | MLE1-12 | unclassified |
|  | OTU00288 | 0.027 | Cyanobacteria | 4C0d-2 | MLE1-12 | unclassified |
| **AAQQ1** | OTU00150 | 0.049 | Proteobacteria | Gammaproteobacteria | unclassified | unclassified |
| **AAQQ2** | OTU00004 | 0.005 | Proteobacteria | Gammaproteobacteria | Oceanospirillales | *Alcanivorax* |
|  | OTU00055 | 0.014 | Proteobacteria | Alphaproteobacteria | Rhodobacterales | *Paracoccus* |
|  | OTU00164 | 0.009 | Proteobacteria | Alphaproteobacteria | Sphingomonadales | unclassified |
| **AAQQ3** | OTU00137 | 0.038 | Proteobacteria | unclassified | unclassified | unclassified |
|  | OTU00217 | 0.010 | Proteobacteria | Gammaproteobacteria | Alteromonadales | *Alteromonas* |
| **AAQQ1 / AAQQ2 / AAQQ3 / QQ-2** | OTU00002 | 0.001 | Proteobacteria | Gammaproteobacteria | Enterobacteriales | unclassified |
|  | OTU00020 | 0.003 | Bacteroidetes | Flavobacteriia | Flavobacteriales | unclassified |
|  | OTU00041 | 0.003 | Bacteroidetes | Flavobacteriia | Flavobacteriales | unclassified |
|  | OTU00044 | 0.003 | Bacteroidetes | Flavobacteriia | Flavobacteriales | unclassified |
|  | OTU00047 | 0.003 | Proteobacteria | Alphaproteobacteria | Rhodobacterales | unclassified |
|  | OTU00057 | 0.001 | Planctomycetes | Phycisphaerae | Phycisphaerales | unclassified |
|  | OTU00064 | 0.001 | Bacteroidetes | Flavobacteriia | Flavobacteriales | unclassified |
|  | OTU00065 | 0.004 | Proteobacteria | Betaproteobacteria | Nitrosomonadales | unclassified |
|  | OTU00067 | 0.001 | Proteobacteria | unclassified | unclassified | unclassified |
|  | OTU00070 | 0.003 | Bacteroidetes | Flavobacteriia | Flavobacteriales | *Winogradskyella* |
|  | OTU00077 | 0.023 | Proteobacteria | Alphaproteobacteria | Rhodobacterales | unclassified |
|  | OTU00078 | 0.003 | Proteobacteria | Gammaproteobacteria | Thiotrichales | unclassified |
|  | OTU00079 | 0.001 | Proteobacteria | Alphaproteobacteria | Rhodobacterales | unclassified |
|  | OTU00087 | 0.003 | Proteobacteria | Alphaproteobacteria | Rhodobacterales | unclassified |
|  | OTU00092 | 0.003 | Proteobacteria | unclassified | unclassified | unclassified |
|  | OTU00094 | 0.001 | Proteobacteria | Gammaproteobacteria | unclassified | unclassified |
|  | OTU00096 | 0.04 | Bacteroidetes | Flavobacteriia | Flavobacteriales | unclassified |
|  | OTU00097 | 0.004 | Proteobacteria | Gammaproteobacteria | Chromatiales | unclassified |
|  | OTU00101 | 0.003 | Proteobacteria | Betaproteobacteria | Methylophilales | *Methylotenera* |
|  | OTU00108 | 0.003 | Proteobacteria | Alphaproteobacteria | Rhodobacterales | unclassified |
|  | OTU00110 | 0.003 | Bacteroidetes | Flavobacteriia | Flavobacteriales | unclassified |
|  | OTU00111 | 0.004 | Bacteroidetes | Flavobacteriia | Flavobacteriales | *Ulvibacter* |
|  | OTU00117 | 0.001 | unclassified | unclassified | unclassified | unclassified |
|  | OTU00121 | 0.003 | Proteobacteria | Alphaproteobacteria | unclassified | unclassified |
|  | OTU00130 | 0.008 | Proteobacteria | Betaproteobacteria | Nitrosomonadales | unclassified |
|  | OTU00131 | 0.003 | Proteobacteria | Gammaproteobacteria | Alteromonadales | *Shewanella* |
|  | OTU00136 | 0.005 | Proteobacteria | unclassified | unclassified | unclassified |
|  | OTU00142 | 0.004 | Proteobacteria | Gammaproteobacteria | Oceanospirillales | unclassified |
|  | OTU00143 | 0.033 | Bacteroidetes | unclassified | unclassified | unclassified |
|  | OTU00150 | 0.005 | Proteobacteria | Gammaproteobacteria | unclassified | unclassified |
|  | OTU00153 | 0.039 | Proteobacteria | Alphaproteobacteria | unclassified | unclassified |
|  | OTU00161 | 0.001 | Proteobacteria | Gammaproteobacteria | unclassified | unclassified |
|  | OTU00164 | 0.013 | Proteobacteria | Alphaproteobacteria | Sphingomonadales | unclassified |
|  | OTU00169 | 0.003 | Firmicutes | Bacilli | Lactobacillales | *Streptococcus* |
|  | OTU00172 | 0.001 | Proteobacteria | Alphaproteobacteria | Rhizobiales | *Filomicrobium* |
|  | OTU00177 | 0.001 | Proteobacteria | Alphaproteobacteria | unclassified | unclassified |
|  | OTU00181 | 0.009 | Proteobacteria | unclassified | unclassified | unclassified |
|  | OTU00186 | 0.001 | unclassified | unclassified | unclassified | unclassified |
|  | OTU00195 | 0.04 | Proteobacteria | Alphaproteobacteria | Rhizobiales | unclassified |
|  | OTU00203 | 0.004 | Proteobacteria | Gammaproteobacteria | unclassified | unclassified |
|  | OTU00219 | 0.009 | Bacteroidetes | Cytophagia | Cytophagales | unclassified |
|  | OTU00220 | 0.001 | Proteobacteria | Gammaproteobacteria | HTCC2188 | unclassified |
|  | OTU00221 | 0.036 | Proteobacteria | Gammaproteobacteria | unclassified | unclassified |
|  | OTU00224 | 0.042 | Proteobacteria | Alphaproteobacteria | Rhodobacterales | *Pseudoruegeria* |
|  | OTU00225 | 0.013 | Proteobacteria | Gammaproteobacteria | Legionellales | unclassified |
|  | OTU00230 | 0.025 | Bacteroidetes | Cytophagia | Cytophagales | *Reichenbachiella* |
|  | OTU00231 | 0.013 | Proteobacteria | Alphaproteobacteria | Rhodobacterales | unclassified |
|  | OTU00236 | 0.018 | Proteobacteria | Gammaproteobacteria | unclassified | unclassified |
|  | OTU00238 | 0.01 | Proteobacteria | Gammaproteobacteria | Legionellales | unclassified |
|  | OTU00259 | 0.005 | Proteobacteria | Gammaproteobacteria | unclassified | unclassified |

**Tab. S5: Negative indicator OTUs.** Significant indicator OTUs are listed, which are absent in control polyps.

| **Indicator OTU** | **P-value** | **Taxonomy** | | | |
| --- | --- | --- | --- | --- | --- |
|  |  | **Phylum** | **Class** | **Order** | **Genus** |
| OTU00002 | 0.0019 | Proteobacteria | Gammaproteobacteria | Enterobacteriales | unclassified |
| OTU00003 | 0.0017 | Proteobacteria | Alphaproteobacteria | Rhodobacterales | *Ruegeria* |
| OTU00013 | 0.0017 | Proteobacteria | unclassified | unclassified | unclassified |
| OTU00015 | 0.0034 | Proteobacteria | Alphaproteobacteria | Rhodobacterales | *Donghicola* |
| OTU00023 | 0.0072 | Proteobacteria | Alphaproteobacteria | Rhodobacterales | unclassified |
| OTU00024 | 0.0012 | Proteobacteria | Alphaproteobacteria | Rhodobacterales | *Roseovarius* |
| OTU00054 | 0.0038 | Proteobacteria | Gammaproteobacteria | unclassified | unclassified |

**Table S6: Bacterial strains and plasmids used in this study.**

| **Plasmid** | **Description** | **Reference** |
| --- | --- | --- |
| pCC1FOS™ | Fosmid | Epicentre, Madison, USA |
| pCR®II-TOPO® | TA-cloning vector | Life Technologies, Karlsruhe, Germany |
| pDrive | Cloning vector | Qiagen, Hilden, Germany |
| pMAL-c2X | Cloning vector encoding maltose binding protein | NEB, Frankfurt a.M., Germany |
| pRS488 | *ccdB* under transcriptional control of the *luxI* promoter | [^1^](file:///C:\Users\Nancy%20E25\Desktop\QQ_Aurelia\NatureScientificReports\revision\Supplement\Tab.%20S5.docx#_ENREF_1) |
| pRS489 | *ccdB* under transcriptional control of the *lsrA* promoter | [^1^](file:///C:\Users\Nancy%20E25\Desktop\QQ_Aurelia\NatureScientificReports\revision\Supplement\Tab.%20S5.docx#_ENREF_1) |
| pRS610 | *aaqq1* fused to *malE* in pMAL-c2X | This study |
| pRS748 | *aaqq2* fused to *malE* in pMAL-c2X | This study |
| pRS753 | *aaqq3* fused to *malE* in pMAL-c2X | This study |
| **Strain** | **Description** | **Reference** |
| *E. coli* DH5α | F-ø80d*lac*ZΔM15 *rec*A1 Δ(*lac*ZYAA*rg*F)  U169*deo*R *end*A1 *hsd*R17(rk^-^mk^+^) *pho*A *sup*E44 λ- *thi*-1 *gyr*A96 *rel*A1 | [^2^](file:///C:\Users\Nancy%20E25\Desktop\QQ_Aurelia\NatureScientificReports\revision\Supplement\Tab.%20S5.docx#_ENREF_2) |
| *E. coli* EPI300™-T1^R^ | F- mcrA Δ(*mrr-hsd*RMS-*mcr*BC) ø80d*lac*Z ΔM15 Δ*lac*X74 *rec*A1 *end*A1 *ara*D139 Δ(*ara,leu*)7697 *gal*U *gal*Kλ- *rps*L *nup*G *trf*A *ton*A *dhfr* | Epicenter, Madison, USA |
| *E. coli* BL21 (DE3) | F^–^ ompT gal dcm lon hsdS_B_ (r_B_^-^ m_B_^-^) λ(DE3 [lacI lacUV5-T7 gene 1 ind1 sam7 nin5]) | [^3^](file:///C:\Users\Nancy%20E25\Desktop\QQ_Aurelia\NatureScientificReports\revision\Supplement\Tab.%20S5.docx#_ENREF_3) |
| *Klebsiella oxytoca* M5aI | DSM7342 | DSMZ, Braunschweig, Germany |
| *Vibrio parahaemolyticus* | isolated from *A. aurita* polyp | Accession No. JX287307 |
| *Pseudoalteromonas tunicata* | isolated from *A. aurita* medusa | Accession No. JX287299 |
| AI1-QQ.1 | reporter strain to identify AHL-QQ compounds | [^1^](file:///C:\Users\Nancy%20E25\Desktop\QQ_Aurelia\NatureScientificReports\revision\Supplement\Tab.%20S5.docx#_ENREF_1) |
| AI2-QQ.1 | reporter strain to identify AI-2-QQ compounds | [^1^](file:///C:\Users\Nancy%20E25\Desktop\QQ_Aurelia\NatureScientificReports\revision\Supplement\Tab.%20S5.docx#_ENREF_1) |
| pZErO-2 control | *E. coli* XL1-Blue/pZErO-2; control strain | [^1^](file:///C:\Users\Nancy%20E25\Desktop\QQ_Aurelia\NatureScientificReports\revision\Supplement\Tab.%20S5.docx#_ENREF_1) |

1 Weiland-Bräuer, N., Pinnow, N. & Schmitz, R. A. Novel Reporter for Identification of Interference with Acyl Homoserine Lactone and Autoinducer-2 Quorum Sensing. *Applied and environmental microbiology* **81**, 1477-1489 (2015).

2 Hanahan, D. Studies on transformation of *Escherichia coli* with plasmids. *Journal of molecular biology* **166**, 557-580 (1983).

3 Studier, F. W. & Moffatt, B. A. Use of bacteriophage T7 RNA polymerase to direct selective high-level expression of cloned genes. *Journal of Molecular Biology* **189**, 113-130, doi:0022-2836(86)90385-2 [pii] (1986).

**Table S7: Primers used in this study.**

| **Primer designation** | **Primer sequence (5´-3´)** | **Characterization** | **Reference** |
| --- | --- | --- | --- |
| AAQQ1_PCRfor_ | GCTGTAGCCGTTGTATCAGG | amplification of *aaqq1* | This study |
| AAQQ1_PCRrev_ | ACCGTTCAGCATTTGGTTG | amplification of *aaqq1* | This study |
| AAQQ2_PCRfor_ | CGTCGGAACGAGAGATGTTG | amplification of *aaqq2* | This study |
| AAQQ2_PCRrev_ | TGGTCCTGGTGTCAAAGGTG | amplification of *aaqq2* | This study |
| AAQQ3_PCRfor_ | GATTGCTGCAGAAATGAGAG | amplification of *aaqq3* | This study |
| AAQQ3_PCRrev_ | GGAATGGTGATGTTTGTGTC | amplification of *aaqq3* | This study |
| AAQQ1_RTfor_ | GGGGTTAAACCAATCCATC | amplification of *aaqq1*  in qRT-PCR | This study |
| AAQQ1_RTrev_ | AATCTAGGTGCGGTCTTACG | amplification of *aaqq1*  in qRT-PCR | This study |
| AAQQ2_RTfor_ | CGGCGTAGCTGCGAGTAAC | amplification of *aaqq2*  in qRT-PCR | This study |
| AAQQ2_RTrev_ | TGGTCCTGGTGTCAAAGGTG | amplification of *aaqq2*  in qRT-PCR | This study |
| AAQQ3_RTfor_ | GTGCAGATCATTGGAGTTTC | amplification of *aaqq3*  in qRT-PCR | This study |
| AAQQ3_RTrev_ | GCGGATAGCTGATGTTACTG | amplification of *aaqq3*  in qRT-PCR | This study |
| AAQQ1_for_ | GAATCGCTTCAATGAAATTTAATACTGAGAA | amplification of *aaqq1* for cloning in pMAL-c2X | This study |
| AAQQ1_rev_ | GAATTCCTATTAGGTGAGACTATAGA | amplification of *aaqq1* for cloning in pMAL-c2X | This study |
| AAQQ2_for_ | GAATCTATTCTATGATCGGCGTCGGAACGAG | amplification of *aaqq2* for cloning in pMAL-c2X | This study |
| AAQQ2_rev_ | AAGCTTTTAAGAATCATCTTCGTTAA | amplification of *aaqq2* for cloning in pMAL-c2X | This study |
| AAQQ3_for_ | GAATCTATTCTATGAAATCACAAACAGTGAT | amplification of *aaqq3* for cloning in pMAL-c2X | This study |
| AAQQ3_rev_ | GGATCCCTACCCCTTAGACAGATGAAA | amplification of *aaqq3* for cloning in pMAL-c2X | This study |
| actin_3F_ | GGTGATGAAGATGTAGCAGCTCG | α-actin housekeeping gene, control for PCR to check for DNA contamination | This study |
| actin_336RV_ | GTTGAGTGGGGCTTCTGTGAGC | α-actin housekeeping gene, control for PCR to check for DNA contamination | This study |
| actin_204for_ | AGCGATCTCCTTCTGCATTC | β-actin housekeeping gene, control for qRT-PCR with C_t_ value 17-18 | [^1^](#_ENREF_1)^,^[^2^](#_ENREF_2) |
| actin_501rev_ | CGCACTCGACTTTGAACAAG | β-actin housekeeping gene, control for qRT-PCR with C_t_ value 17-18 | [^1^](#_ENREF_1)^,^[^2^](#_ENREF_2) |
| M13(-20)for | GTAAAACGACGGCCAGT | standard primer | - |
| M13(-24)rev | AACAGCTATGACCATG | standard primer | - |

1 Wan, Q., Whang, I., Choi, C. Y., Lee, J. S. & Lee, J. Validation of housekeeping genes as internal controls for studying biomarkers of endocrine-disrupting chemicals in disk abalone by real-time PCR. *Comp Biochem Physiol C Toxicol Pharmacol* **153**, 259-268 (2011).

2 Romero, A. *et al.* Individual sequence variability and functional activities of fibrinogen-related proteins (FREPs) in the Mediterranean mussel (*Mytilus galloprovincialis*) suggest ancient and complex immune recognition models in invertebrates. *Developmental and comparative immunology* **35**, 334-344 (2011).
