# Supplementary material for: Potential role of host-derived quorum quenching in modulating bacterial colonization in the moon jellyfish Aurelia aurita
Source: Sci Rep. 2019 Jan 10;9:34. doi: 10.1038/s41598-018-37321-z (PMC6328592; doi:10.1038/s41598-018-37321-z)
Supplement: Supplementary file 2 — Dataset 1 [file 41598_2018_37321_MOESM2_ESM.docx]

# Potential role of host-derived quorum quenching in modulating bacterial colonization in the moon jellyfish *Aurelia aurita*

Nancy Weiland-Bräuer, Martin A. Fischer, Nicole Pinnow, and Ruth A. Schmitz*

**Supplemental dataset**

>full length insert of fosmid 2

TTGAAAAATCACCAAAATTTGATTATATGGCCGATCTTTAAATTTCTTTCTTTGGCAAGTTGATCAAAGACCACATTATTTCCCCAGGTATAGCTGATCTTGTGATTGGTTTTAAAATTAGCAACTTTAGCTCGTTTTATGGCTGCCTGGTTTTGAAGCATACTGCAAAAGAGCACTGATGATAACCAGATTAATTTCTTTGTGTATATTTGGCTTTTAGTTTGGTCATAAATGACAGAAAGAAACGTGAACTATCATTATAAACTTGAAGGATAATGAAAACTTTCAAAATAATGGCTCTTTGGCAAGATTTACGATGTTCATTAAAGAGTATAGCGCAGAATAAGGTTTTTCAACCAGTCATTTTATACCCTTTAATAACGAAAACAAACATGTAAATTATGTAAACAAACATGTTTTCATCTTTAAATATATTTGACTCATTGACAAAAGAGACCAAAAAGAAGAATATTTATGATAGATTTGATATTTGAGTTTTGGTGATTTTTTAAACAGAGTACTTTTTTGGGACACCCGGTACCTTCCCCCTAGTAGGGGGGTACAGGGTGCGGGATTTTAAAAAGATTGGCAGTCTTTTGGTGAAATTCCTCATGAAAATGGGTATGTATGTATGTATGGTTTTGCCATAAAATTAATTACAATTTAAATTTAAGAAAAAAGAAAATGATCTATAGTTATATGTATACAAAATTTACACGCAAAAGCTCAAAATGTGGTCCAAAAACACCCCAGATTAAAAATGGCAGGGATAGCATTAAAAAGTTCAATGCAATCTTGTTTCCATAATGGTCCCTCTTTGGTACAAAGTTGAATTTTGGCAATTTGAAGAGGATACCTTTCTGAAAATTAAGTGGCCAACCCCCCCATTCCACCACAAGTTTTGTACCCCCTTGTGACAATTCTCTCGTAAAATCGATAATAACACAACTCAAAAACGTTGGTTCTTGTTCTATGAATGCTCAAAAGTACCTTGACTAGGCTTTCAAATCTGCTAAAAAATGCTTAGATTTTTTCATTTTATGCTATTAACACCCACTGAGAACCATAGAAAATTATTAATTATGAATCCATATGTTGGAGATCCCTTGGAGTAGGGTACATGAATTTAACCCTGGTTTAACAGTTTGTGACAAAACCTTTGCTGATTCATAAACTACTGACTCAAAACCTTGAAGTAAGCCCTTTTGTCAGAGCCCTTCATCTTGTCAACCACATCTGGTAATTGTCATAGTGTTTTTTTAATCAGCTTTTGCTTGGGTATGGGATGAAAGTAACAAGTCACATGATGTAATGGTCTGCAATCAAGGCAAAGAAGCATATTTCCATCCAGATTACAGTTGTGGTACCGCAGCAGTGAGAGGCACACACGCATTAACATATGGATGGGAACATTATTGGGAAGTGAAAATGGCTAGTGCAGTCTATGGCACTGATATGGTATTGTTGTGGCACTGGTGTCATCAATTTCTTATTTTTAATTAAAATTTATTTTTAGCAATCTCTTCAAAATGTGTAGAACGAGAAAAGAATGATAAACAATTTTATAACACGCGGAGGTTTTTTAAAAATGTTATTCTGACTACAGAAGATGTTTCACCATTCACTATTTTAAAATGTTTCTAAATAGAACCTTTGCCTCTTAATTTAACTGTTTGTAGCTAGAATTTATTTTGATTTATTTTAGATGATCGGCGTCGGAACGAGAGATGTTGATTTGAATAGATATAAATCACAATTTTGCAGCATGCTAGGACGTGATTCGGAAAGTTGGGGATTATCATACTTTGGAACTTTCCAAAATGGCGGAAAAACAAAAGAATTCACAGATAAATTTGAACGAGGTACCGTAATTGGAATACATTTGGATATGTGGAAAGGTACACTGTCTTTCTTTCGGGATGGAGATCTCCTCGGCGTAGCTGCGAGTAACTTACAAGGAAAGATTCTTTACCCTATAATTGCATCGACCGCTGCGAGGACGCGCATGAAGCTGGAATGCAGTCATAGTACCGTATTTTCGTTACAATATTTATGTGTGAAAGAAATAAGCAAACACATCGATCATACTGCTGAAGCTGTGGAGGAGCTGCCACTTTCTAAAGGACTCAAGATGTACTTGCATCGTCATGTAAAATGGTTGTCTCATGTAAATAACGCACCTTTGACACCAGGACCAAAACGGAAGAAACAAAGAAGATCAAATTTGTACAAGAATGAGGAAGATATTGAGCTGATTAACGAAGATGATTCTTAAATGATTAATATATGAAATAATAAAGTAAGATTGAATATATTATAGAGTTTTTCTAACTTTTCATTTTACATTTTCGATTTTTAATTACAGCTTTTAGATAAACATGCAAAGATAGGCCAAAGAGAACGGCATAAGCTCGAGGTAAAGTGTGAATTGTCCAATGCTAGGATTATTAATTTCATGAAAATGAATGCTTCCTGAGAATACTGCAGCTAGTTCAAGGATATTTTTAACGCTGAACTGAATTTCAAATTTACACAATTGAGCGTACATGACATGAAGTCCAGAGAGTTGCAGTCATATTAAGATGTCCCCTCCCTCAGAAGCTTTGCTCGACCATTGGCCCACTTCTCTATCCGAACTGGCATATGTCATTCTATTGCATATACAAGCACGAGGTTGTTCATATGAGGTAGGATGTTGTTTACATCTTGCGAGGAAGAAATGTCAAGTCATTGGTTGTTTCAGCGTCATCGCCTTTTATTTGTATCTATTTCCATCTTCTCTTAATAACAATGTACAATATAAACAAAGGTCTACTTTATCCCGATGAAAACAAATTTAGTGATTGCAAATTTCGTTCTTGAAGTATAAGGATTCATTTTGCTGCCTTTTTGGCGAGGAAAGAAAGTAGCTAGCCGTTGATTTTGTAGACATAGTTGTAAATAATTTTTAATCTTTTAACTTTAAGACTTCACGAGAATCTCTAAATAATGCACCATGGCATGAAATAGAATTAAATCTGATAGAACGTCTGCAGTGATAATATTACTTTCATAAGAATCCAACAAGGTTTCAAACAACCACTGCAGTGTTGATAAAACTTTGCTGTCAGTGAGTTGATTCGCTTTTGTTAGCCTACTTTATCAATCAGAGTTTGCCATGTCCAATAGAAAGATTTAAGAAACCATAACGTTTTTTACACTAAATGTAGAATATGAGAAGAAATTGTCAATGAACTAGAGAAGTCTGTATATAAACACTTTATAATTCCGCTGTATGTACTCTATGCTAAATAGTGCTCAATAACAATAGATTAAAAGCAACAGACTAATGCGAATGCAAATTATGTATAAATGAAAAATTGGACATATAGTCTATCAAGAAAGAAACAGTTGAACAAATTAATAATTCGTCTTTTGTATTTATTTAACATTTTGTTTTTGCTAAATGTTATAAAGAATTTAATTATGTTGTTTGTATCTTGCTTATATCTCTCAAACTGACAATTTTGATTTTGTTTTGCTTTGGAGATGTACGTACGTTAGGTTCAATCAAAAACCTCACGCAAAAGAACTCAACGTTTACGTTTATGCATTAACTTGATTTAATCGAGCCAAGAAAGCGTTTTAACTCTTTAAAGTTGCTCTTGATGGTTTTCTTTACTACGAATTTGAAAATTATTCGAAATCATGAGTCAGTTCATTTCTGTGAATGACGATTTGTGCACTAGGCTAGTGTTCAAATCTATATTTCCCAGGCCCAGAAAATGATATCTAAGGGTGTGTTTGATTGGTAGTTCCAAACTTCCAAACCTACCAAATTCTGAAAACAGTAGACTTCGCAGAGACGGAAATATAGTTCCCAATCAAACACAAAAATCTAAACAGCAGGTTTTGGATTCACCATTGCGATTCGCTAATGAAAACAAGGATCTCTCCAAAGTCTGCCAAACCTTCTGCCTTTTGGAGCAGAATGTTCACAAGGCCTTCCAAACCTATCAAACCTACCGTTTTGAGAATTTCGGAGTCAATTCCGAGTCTACCAAATCTACCGTTTTCAAAATTGGTGCCTATCAAACACACCCTAAATTTGCATTCATAGCGGTTTTACGCAATTTGATTGGTCCCTTTGGAGAAAGTGTGTCATTTTTATAAGTATGTCATTTTTTAAGCCAATCAAGATTTTTAAAAGTTGGTTTGCAATCTCAGACCAGCGCAGGGGGTAGCAGTAGATTTATTACAATCGTCGTAGTTTCGCTCCTTGATGGACTGATAATCGCGGATGACCGTCATCCAATTCAATAAACAAAGTGGAAACTGGGACTGAGGTAATCAATCTAGGGTAGCAGTCTCGTTTTCGTTGCTTATTTTTTGATAGGTTTCTTTGAAAAATGGTAATGGCGGATGATAAAACGTGAAGCTGTGGGAAACGCGAAGAGCAAGAAAGGCAAAGACAGGTTACTTTCTAAGTATATTTATAAATTGATATGACTTATTTATAACGAGGTAATATAAAAAAGGAAATTATGATTACATTTTGTAGAATTTAGCGCGAAAAATAGCATCAAAACAAAAGTAAGATTTGTTTTGAAGAAGAGGGCCTAGCCCCCTAGCCTAATAAACTGTGAAATAAACTAAAAATATAATGAGAAAAAACTGTAAAATAGACTTCTTCAACCTCGTAGCATCTACATCTGCATATTTCTATTCCATGAAGGTTTCCATTGATTACTGCTCCTCAGAGTGAATTTTTAAACAAGCTTCTACTGTCATGGGGAAAGGTAGAGATCGAGATAGAGGGCAGAAGAAGAGAAAAGACAAATCAAGTGAAAAGCATGGATTTGAGAGTTTTGAAAGATGTGGAGATTCTTTGCCATCAGTTGGTGCGGAAGGGTACACTCATGAAGGTTATTTGCACAGTTTTCTTATAATAATATCTTTCTTCAATTTTCTGATATCTACTGTACAAATTCAGTATTTGACCCAGTACTGAAAGTTTCTTAAAATCCTCACTATCTGAGTGCTCAAAGTTTCTTAATGGTTTCTTAGGAGTCAAATTCTTAATGTTTACATCTTGTGAACATTAATTCCAAAACAAAAATTTCACGAAGCAATGACACTGTCTGGACCATATTCATATCTTCTGACTGAGTAATATTCATTGCAAAAACAAGTGAACATTAGTGAAAATATGCTATGGATTTCATGAAATACTACAAAAAAATGTCTTGAATACTTTAGTCCCAGCACTGAAGCTTCTTAATTTTTTAGTGATATGAGAGTGGAGTTTCTTGTAAAGGGCTTCTTATGAAAAGTATGTCTATTTTATAATCCATGCGTGTAAATCTAATGGAGGGCAATGATATTTAATTCCTCTTCAATATTTCTGCATGCTTTTTAAATTTTCAGATGCTGCTACACTATCAGGCAATAAATTTGGTTTTCCTCTGGCTATGTGGGTGAGTGTAAAACAGTGACTGCGTCAATTATTTAATTTTGTTAATTTATAATAAAGGGCTAAGAATATCAGAGTTGAAGGTGAAGGTGAAGTTGCGGTTTTTAGCTTTAAACGCTGGGATCACAGAAGTAGAGAAGACCTAATCGAGTCCGCGACCCTTTGTCATGACACTCAAGTGGTGTGGCTCGTACTGGGATCGAACCCTGTCCGCCTGATTGTTCTGGCTGCAAATTCAGAGGATTGAACCACTGTGCCACAACACCTTCAAGAGTTTCTCTATGGCACTTTTTTGTGATAATTGATGTACACACATCATCCTTTGTTGTAATCTGTAGTATATACTACTTCCATTCAATTTTATTGTAAAATTCTACTTGTTTGTAGCAAAACATGAAAAACTATAATATATGCTGTTGCATTTAGTAACAGAACTATATGCTAAATATTGTTCTCTGACCCAGGTCACAGCAAATTGTTATATAAGAATGAACAGAAAATTTGCTGTGACTTTTGTCAGATGAGATATTTAGCAAAAGTGGTTCCTTTACTCAGCGTAACTTTCAAGTTCAGTCCGGCCCCGGGAGTCAGGCATGAAAGTTATTTTACATAACTCAATATGTTGCCTTGCAGGACCTTGCTCATTGTGACCCAAAGAAATGCACTGGACAAAAACTGAGTCGATTTGGTTTCTTGAAGATATTACGATTAGGACACAAATTTAATGGCATTGTTTTAAGCCCTCTTGGAAGGCAATATGTGTCACCGGCAGACAAAGAAATTGTGAAGGAGCATGGAGCTGCAGTGATAGATTGTTCATGGGCTAAATTAAATGAAACACCATTTGGTAAAATGAAAGCGAGGCATCCAAGGCTTTTACCCCATTTGGTTGCTGCTAATACGATTAACTATGGAAAACCTTCAAAATTATCTTGTGTGGAAGCATTTGCTGCTCTTTTCTATATTACAGGTGAGGTTTCACTTTTATGTAAGAAAATCTTTACGAAAAATCCTCAGAGACAATCTATAATCTAACTAATAATCGTATGCTCCCTTCAATAAATATGAATTAGATAAATAATTTGTAAAAATATCGAATGCTTGCTGTAACCTTTCGCTTTATGATGAGGCAAAAGTGAGACGTTAGCAAGAATTTATGTATTTTTGAAATTCATTATAATCCAACTATGTGTATTTCCAGAAGCATCACACCAGGAATTCTTATGGTTGGAAAGAAATATCAGAATCCCTTTGGAATTGAAATTGCAGGTTTTGCCTTGAATGGAAAGCGATTTTATCAAATGAAATGTCCCTAAAATTATCCTCATCCTTCCAAAGGAAACATGTTTTGAATTTCAGCATTCCCACATCAGAGTACTTTCCACGAACAGTTGCAAAATAATTGTGCTATATCTGTGAGAAAGCGCTCTGCTCTTCATTTACTCCTTTATTGGAAATAAACCTTTCAGTCTTGTCATGGAAGATTTTGGGGGGAAACACTTGTCGGTTGATTCTTGTAAATGTTGAAAAATGTTTCTGTAGCGACCAAAGCCTCGTACTTTTATCTTGGTGGATGTTCTAGTCAATTCAAGTTGGATTTACAGTTGAATCCTGAAATAACGAGCCACCGATATATATAACGAACTTCACGCTTAAGGAACTAAATGTTTGATCCGTTGAATGGCAATTTTTTGATAAATTCTGTTGGTTTAACGAACTCCCGATAGAACGAATTTTGTTCAGGTTCCCCTCAGAGTTTGTTATACCAGGATTCATCTGTATATTCGTGAATAGCTAAATTCTCTGGCTTGCATATTCCGATTATTAACAAGCCTGCTACATTGGTCATTTTAAGCAAAGGTGAAGAGAAAATGAATAAGCTTGAGAGGAGTGCCTTTTACAGTTCTTGTCACACTGCTAATTACCCTCCATTATCATCTCAACGATCAAAACGCCCTTCTATGTATTTCCTTTTAAATCTAGGTTTCTCGGATGTTGCTGAAACACTCTTACAAAAATTCAAATGGGGGGAAGGTTTCTACGAATTAAACCGGTAAGAGAACCGACAAATTTTATTCGTTTAAACCTTACCCTTTTTTATTAAGTTGTAAATTGACTTGTTCTGAAACCTTTCATTTTGTTAATTTAAGGTAGCAGTTAACCCTTAGGAGCCAAAAACTTTACTAGCAAATAACTACAATTCCTTGCATAGTATTTCCATGAAATTTTCAGAAAAGCTTGGAATGGTAGATAGAATTAGAATTTTGTCAAAAAAAAATTTCTGAAGACAATCGTTTCCATGGAAACAGTCATTATTGTTTCCGAAAATTTTAACAAAAATGCCATTTATTCTGGCCCAGTATGAGATATTTGGCTGAAACTCCTACTAAGGGTGTGCTTTATAATGTTCTATGCAATGAATTATCAATTTTGCGAAATTTTGTTACAAATTAGGCAACTGTCACCAAAGAAAAGTCTCCATGGCAACCGGGCGTGGCTTCAAAAATTTTCAAAATTTACATAATTTATTGCAAAGAACAACATTTGAGACCTCTTTTGTGAAAATCTCATTAAAATATCAACAATGGTGTCAAATTGGCAAAGGATATTGCTTTCACAGTTGGCAAATACAGCATTTTGAGAAAAACAGCCTTAAAGTTGAACAAGTGGTTTACATTGGGTTTCCATCAAATGGTGAATTATAAACAAGGTTTTACAGCCCATAAATTATTCTAGCAACTATTTTTTTCTACCAGTTAAGTCCATTTGTAGTGTCTTCCTTTTGTATGGTCCTTCAACTGGGTCTTGTTCTGCATTTTTATACCACTAATGCTACTATTTGCTGGAACTACATGTACTGAACCAGATCTACTTTCAGGTCTGCGTAGCCTTTGCACATTGACGATTGACTTGAAACCCCCTGTACCACCAGTCTTTTAGTGCTTGTTGAAGAAAAGAATTTCCTCCATATCTCCTGCACTTCAAGGATAACTATCCTGCAAGTCCAGCTCTCCCTCTTTGTTATGTTGTAGTAGTTAGAGTTAGTATTCCAGTCTTGCAAGCAAAACAGACAGATCTTGCATCTTGAAGAATCCTTTTGTTGATGTTGTGTTTGCCAGTGGGCGTGGCATACAGAACATGCATGCTGTCCTGTTGCATCCTTAACTTCCTGGCAAAACTTGGTATATTCTCAAATGGGCTACTAGAATGTTCTGTGCAACCTACTTTGATTTGTATCTTCAACTTCTCCAACTTGGATTGTATTTACTATTTGCCTTCACCTTGTTTGGTGTGGTGGAGTACGAGTTTATAAGACATCACATCACATCAGTCACGTCACTTTTAGGCCCGGCTGTTGTAGCCCCTAATACTTCGTTTTGTGCCTTCTCTACCATTGCTGCTCGTTCTCTATCTACTTGTTACTTATTTTGTTCTTGATAACCTTTTCTTCCTTTCCTTACTCCTAAACATTTGGTAACTTTTGGCCTTCAGGTCTGCATCACTTTAGTTCTCCCCTCCATTTCTTCATTCCACAAAAAGCACTTCTTTGCGCTGTTGTTGTTGCCCAAAGAAAGGAGTCATGGGATTGGAAAAGACGGAATGGAGCCCAAGAGCATGAAACGAGGGATTTTTAACCAATTTCTGCACAAAATTTTGGAAAAACCTCCTTAGTAACCAAAAAAATAGTTTGTTGCTAAGGAAAATTGGGATGACCTCTTCATATAAATATTAATTTAACGGGGATATGAACATCTATATAGGCTTGGAGGCTGCTTTATCTTTAAAAGGAATAAATAAACTATTGAATAACACAAATTTCAAAATTTGGCTCAAAATTTCACATCTCTCAAATCCTAAGGGTTAACTGCTACCTTAAATATGATTCTTTACCTATTGATAGGAAGAGTGAAAACTATTTTTATTGCACATTTCAATTTTGTTCAGGGAACTCCTCGAAAAATATTCTCAATGCAAGAACGCAAAAGAAATCATAGTTGCAGAAAAATGTTGGTTAGAACAATGCGAAGAAGAAAACAGAAAAATGCGAGAAATCGGTAATTATCTTCACCTAGGTCGATTATTTAAATCGTCATCTCATTATTTAAGTTGGAAATTAGCTACAATCAAACCAAGCCGAGAAATCATCGTCACAATTTGATCAATCAAAGATTTGAGCAACTAACCGATTCACTTTCCTCTGCTTTTAACAATTATGGCAGTCAGGCACTACTGGATGAGTCAAAAAGTTATCGGAATATGCGCCAGTTGATAGTAAAAAGGCTCATTTTGATCTGCAAATGTTATTTCATCAAAATTGCTGATGAAATAACAGAATTGCAAAATTGTAGAACATGGAAGCTGTTTTTCAAACAGCTTGAAAAACGACCAGACATTTGGACAAAATTCTGCACAAATTCTGCACAACTTGATCAACTTTTCTTGCCCTTCTTTGAGCATTTTGCAAAAATGCTTCCAGACAATATTGCCCATAACTCTTGCACGATATAACCGATTTCAACGAAATGAATTTCAACATCGAAGTTCACCTTTCTGAGGTAAAACGAAGCATATCACACCGAAATTGGTCGATAATTCTTGGTAGTACATTTCGGCAAAGCTGATAATTTTTAACTCAGTCTGTACGCTGTGAGTTCTTGCATTGATATTAGGGAGTGGTCATTAAGTATGTGCTATGCCGGGGCGGAGGGTATTTAGGAGGGGTACGAGATTTTGTTGGTACTTCAAGATGGGGTACGAAATGAATATTGATATTTCTGAGGGGGTATGAAATCAAAATCACAGTTTTAAAAAAATTGTGCAATAGTGTATATATATAAGTACTTATTCAAATGATAGGGAGGCAGTGTACGAAAAATGATAGCGAGGCTGTCAAGTACCGTCCTGTCGTTTCAGATTATCCTTGTTCTGACTTGGAGTAACAACGCGAGTTTCATTAGAGGCAAGATGGGGTATGAAACAATTTTCAAGAATTACGCAGGGGTATGAAACATTTTAGGAAAAAGTTAGAAGGGGTTTGAAATTTTTCTAAAGATTCTGTTGGTACCCTCCGCCCCGGCATATCACATACTTAATGGCCACTCCCTTACTTCACAATTTAGTGCCAGATTTATTCGAATTCAACATAGCTTCACGTATTGTCTCTTATTGTTGTAGATATGACGGAAGTGGATATGGACAAGGAATATTTTAATCCTAATAGACAGGCGACATCACAGTCATATCGAAGGTAAGATATTGTTTATCAAAGGAAATTTCAAAGAAGAAAATAACACAGAGCATAGAATAGAATTTCACGAAATTTCACTCAGATTTAGAAATCTATCAGGCTTAAATCAGCCGATTCTTTTCAAAATTTTGGAAGATTTTCTGGTGAAGGCCGCTTCAGAAACTTTCAAAACCTGCTGAAAATGATGATCAAATTTCGTGATTTAAATAGAAAACAAAAAAGATATGATTTTGATTTTTAAATGTTTCGATAATATCTGACTAACTAATATGATAGCAAGAAACCAATGAATCAATGTATCATTATTTTAAGGAAGTTTTACAGATTTTGTTTCTTCTCCTTTCAGTGTTTGTTGTTGTCAAATTCCCTAGTTCTTTTGTCTTACTTTTGTTTGTGGTTTTTCCTTGAAGGTATGGAGAACAAGACGACGATGATGATGATGATGATGATGATGATGATGATGATGATGATGATGATGATGATGATGATGATGATGATGATGATGATGATGATGCTGATGCTGATGCTAGTGACACTAAAGACTCTGATGGTGACAAGGACAATGAAGACAATCATAATATTGACGATAACGGTGATCAAAGGGATGAAAGTGACGGCGAATGTGATGAACACAGCGATCAAGAGAAAGTGATTAGTGATTCTGATCTGATTGCAAAGAAGAACAATCACGTGGCTCTCACTCAGAGCATGGAGAAGACGAAAAGGCAATTGATAAACAAATTGCATTTGATGAAAGGGACATAACTAGCCATGACTCTAATGATTTTGTGAGTGGAGAACAGGGCCATGATTTGGCAGATAATGCTACAATTTGAAAACAATGTTTGCTCCAAGTAGGCTTTCATGTCCAGAACTAAACCACTTTTGGTTTCATTTCTTGAGAAGGTGGAAGTGGGTTAACTTCGAAATATACCAAATTTAAACTCATAAATCAGTATTGAGCGAGCAACTGTAGTCATGTAGAGAAGATGTCTTTTTTGAAGAATTTAACTATTTTCAAGAATGATGAGAATGACTTATGCTCCGACAAACAGATCTGCTTTAGGTTCATATTTAAATTCCCACTATCTAAGTATTGGTACTAAAAATTCGGGCGTCGATGAATAGATCACGTACATAATACTCTCTTCTTAAAGGGAGTACAAAGACCAGCATGCTTTGCGCACAGTCAGCGACTTTCTCTGCACAGCTTCCAGAATAACTGACTGTACGAAATTCACAAAAACAAAAACAAACGATGGCAAAGGCTGCTGGTCTTTGTACTCCCTTTAAAGACAAATGACGGATATATAGCGCCTGGTTTAAAAATAGGACAGAAGATGTCTGAGTGGCTTCATTGTCCAGTACATTTGATAATCTCTCGAGGTCATCAAATACAATGGAATACAATAGGCTATATTTGGATTGCCCAGGCCCCTGATTTAAGTCGCCTTATATCTCCGTCATTTGTCTTTAACCTCTTTGTACTGAGCTGCAATGTTTTCTACAAGCTGGGAGGCAAGATTTTATAATTTCATATGCCGTTGGGGAGGAGCGATACTGCAGCCTCTCATTCAAAAATATGCGAAAGCCGCCATTTTGAAAAGGCCTAAGAATTATATTTGATGTGAAGAATAAGTCATGTTTGCTGTGCTGGGAACACGTTTATCGTCATTGATTTCACGAAACTTGCAAGAGATAAAGATTACTGGTAATATATTCAACTCCTCTTAATAAGTATTCTATCACTTCTCGCCCCAGATTGGAAAATTTGACATGCGAGGGAGATTAATCCTTCGATTCGTGCGCTGGCGTGCGCTTTTAATTAATATCTCCGTGATTCTGACCGCGAATGAAGAAGAAATCCATGGCGCATTCTGCAAACTTTCCAATCTGGTTTTCGCCGAGATGTACGAGATGTCTGTCGGGTTAAACAATTGAAGCAAGTGCAGCCATGCCATTTATCCTCTCTATATGATATTCGTTCTTTAATAAGGGGTCGTCCAAAAATCCACTCGTGATCAGTTAACGGCATTTGGCCGCTAACTTTTTGACCCCCTCCCCCCTTCCTTAGCGGACTCTACCTATATTTTTCTATATTGCATGCTGTACTCATATATTGGCCTAAAAATGGTTGACCGTTTATTCATAGGAAAACGACAGACATCATAAGGAACGGTGGCAAAAAACATCGCTACAAATCAATTCTCACCAGTTTCTGTATACAATTTTCAAAGTATTCATCCCATTAAATTCCTTAAGTTCCAACGATGATCGCTTAACGGATTTTGATGAAACCTCCTCCCCCTTAGCGGCCAAATGCCGCTAACTGATCACGAGTGGATTTTTGGACGACCCCTAAATTGATTTTCATGGCTGCTAGCCAGTCTGACTGCAGAGCGTTGGATGTGCACACAACCGTGCGTGGTTTGTAGAGCCATTTTTTCATAAAGAATTTTTAAGACAGTCGCTGAGAGCGATTTTCATACGATACGATCTTTAATTTTCTTAAATGTCGTATGCGTATGGTAAATTTCTCCCCCCCCCCCCAATGGCGTCGCCAGGGTACACCTCACAGAACCAAATTCTTTACTTTGTCCACCCCACTTTTACAGGGCTGGCGACGCCCCTATCCCCCCTTAATGGCCCAATGCTGCTAAAAATATGTAAATCAAAGTTGAATGGGCCCCTTACAGTAAACGAGTTATGAGATTGTCAGTATTGTCAGTTGTTTCATGTTCACATTTAACTTTCAGGTATTCCTTCCATCATTCAAATATGTAATGCAACCAAGAAAGCTGGTGGCCAAGCCAGAAAACAGGGCAAACCAAGAAGGGGCAAAAGAAGAGGAGTAAAGTGTTTAGATGGTATGCACCTTACAATGCAGCATAAAATTTAACCCTTGGATAGATACATATGACTGGAAAATATGGTGTCATGTATAACACTTTTGCTTTGATACAGGTTAAGGTTGTAGGTTTTGAGTTAACTCAATACATTCAGCATAGATACGCGCATTGACTGAACTTATGTACCACTGTGCTGCTATGTATGGTTATATTTTGCCGTTGGTATAACCCACAAAGCATCAAGAAATGCATGTTGTGGTATGGGGTCTGTGGGACGTTTCAAAAGATAAATGTTTCTTTACACCCCATAGTGGAATCGAGGTAGAACTGCCCACCATATGTGTCTGCCAGGCATATTTTTGGTACGCTAGGAGGTATTCCATAGCTTATGGTTCATCTATAAGATTTAGAGATTTTGGGCTTGGCGTATGCCAATGTACTCCAAAATTCAAGGTCTTGTCTGTCATATACTGTACATATGGTGGATTCCTCCTTGATTACGCTATGGTTCACACCATGTCTATAATTTACTTTGGAAACAGTCATGATGTGAAATTTTGAAAATTTTCATAAAATTAGATACACTGACAAGAAAGATCTTTTTGAGTAAATTCAGAAACTGTACTTGTTTGTAGCATTTGAGCAGGTCATTGACGAGTTATGAAAAGTTTGAGTTAAAATTTGGAGTTTTCTTTCAGGTGTGCATGTTAAATCAGGACAGATTCTGGTGAGGCAGCTTGGCTACAAATTTCATCCAGGAAAAAATGTGAGTAAATTTTAACGTAAATAAATGATACTAATAAATAAATAATGATTTAATGTTTCTGTTAATAAGTAGACTTTGCTATGGACATTTTAAGAGTTAGGTTATTTCAACTATGATTTTAACAAAAGCTGCCTCTCTTTCAGAGTATTGAGCTCCTTTGTTTATGATTTACTGTATGTCCTTCCTGGGTATGCAGCCTTTTGGCCTGAACTGAAAACCATCTCCAAAAAGGTATAAAACTAGCAAGGCAGCAAAATTGTTTTTATCAATGAAATGATGATTGCCACTAATAATCAAGGCACTGAATGTACTTCTTGAAACGTTTTTATCAGGCCTCTCATTTAATAGTTTTATAATATACTCATCACTTCTTCGTGCCCTGTTACACCATATTCGTTCAGCTTCTTTCGCTAGATAAAAAACCCTGGCATTCAGACATCCATGGCTGGCTGTTTCTTTTATAAAAACCCCATCCTAATGAAGTAGATAAGATTTATTGTTTTTCCCCGTTAGGTCGCTGCTGGAAGAGATCATACATTATTTGCGTTATGCGAAGGATTTGTCAAATACACAAGTGAACTTTTGCAACCCCAACCATTTAGTCTTTCAAGAAAACCTGTAGATTACGTAGAACGAAACTTTATAAACATTGTGGAAAGACCAAAACCCAGGCGCCTTATTTGTATTAATGAACATGAAATGAAAAACAATACTAATAGTAAACAGTAATACAGTATGTATCTTTGGTGTCATTGTCATTATTTAGAAGAATCTGAGACTGCGTTTATACGATACCGACACAATTTGAAAACGGCATAACATTTTTACGGATGATAGGCCTCTCGTCCACACGAAAACGGCACATTTTTTGCGGGCGGAATTTGAAAACGGAACTCTAACCGGCACAAGTTGAAAACTGCATCGTGTGAATACTCGAAAACGATGAAAATGGAACCTTTTTCAACGCTTTCGAGAAGAGGCTGATTGATTTTTAGACCAAGATCAATTTCTTTGTCGTCGAAATCCAGCCTCTTTGTCGCAGAGGTCACAAAGAGATTGGTACCTTCGAATTTTCTATCGTTTTCAAATCGCGCCTGTATCGTGTGAACACTAGCTAAGATTTAGTACCGTCACATTTTTTGCCGTTTCAAATTGTACCGGCATCGTGTGAACGTAGTCTGAATTGGTTGGCCAACGACCTTCCCGGCTAATCTAAAGTGTCCCCTTGTGTGCCCATTTACTTTTAAATGTAAACTTTTAGCGTTAGAAGTATAACACCGCCGCCTCCATAGAATTGCCCCAAGTCTATTTCCTGAAGTTCATCTGACGGATACCTTTCTTACAGTTCCAGAATCCGCAATTCCAACATCCAGGATCTGTGGGAAAGGGATCGGTCAGACGGACTCTAGGAAATAGACTCAGGGCAAGTGTACTGCCTCTACAAATGCCACATCGTCCCTAATCATGAAACTTATTTTGTTGACATTTTTCCAATAATGTTTGAAGGGAAATAGTGCCCACTTTGAGGATTGAAATGTAACATGGGAGAATGAAACGATAGGAAATGTTGTACCGAGATGGTTGTACTGGTTCCATAGCAAACCGGTATCATTGCCGGGTATCATTGTGTGTTTTGAGTAAGAAAATGTCGTACAGAGTTGAAGCGACGAGAACAAGAAAAGATTATTTCTCGCGTTGAATTGAATTGAAGTAAACTTTAAAATGAAGCAGCAATATGATGAGCATCCTGTTTTTGTCATCGATGTTTCGACTTGTTTGTCTGGGATTTGTCACGTGATCGATCGTAGTTTTGTCATTGTGCCCTTTTGATATTATGCCACTGTTGTTTTAGGCCTATGACTATGCGAACTTCCTTCGGACTTCGGGCTTCCTTCCTTGTTTGCGTTGCTTGCTTTCTTTCTTTCGTATGCAATTTCATTTCAGCGTTTCAGCATTATGTTGATTGTTTGGCAATTTCTGTGCTAACCTTATTTGGTAATTTGGTCACCGACGCTAAAAGAATAAAGATCCTCAACTTCACAAACTTCAGTCAGTCTAATTTGGAAAGAGAAATTTGATTACTGTAGCCTTCTCTGGAAATTCCGATTCTCGTTCCGGTCGTTCTTACACCAAATATCCCCTAGCAGAAAAATGCGAATTGGATCGAAAACTGTAATATTATCAGAGCTGTTGCGTCAAGATTAACTGAACGATTTTTGAAATGAGATTTCAAGATTTCATTATCAAACAATAGCCTAGTACATGTTATTATGTATACTTTAAACAGCTATCGAATGAAGCAAACAAGAACAATAAATTTATTAGCAAAACAACAACGAAGACACTAAATCATACAATGTTATATACATGATAAGAAGTCGATGACAAACAGAACAATGAATTTATTAACGGAAAATCAACAATAACAACAATCGAATCATGTTATACTATAAACGGTCATGACTGAGTTTCATTTGATATGTCCTTATTTTTGTAGGTGTTTGATAAACATTGGTCTATAAAGTCATAAATGGGTTAGGTACCATTAAGTTCTGATGTTTTCATAATGAAGATAAATCTTTTTTTACCATCCATATGTTGAAACGTAGGATATCGTTTGGTAATCTCCTGCAAGAACTGAAATCTTAAATTTTCGTTCAATTTGCATGTCATCAAACAATGTACCTCAGTGGAGTTACATACGTGACATACTCGTTCTTCCGGTTTCCTTTTTGGTCTATGATATCTCCCAATCTCTATTTCTAGTTTGTGGTCAGACAGTCTAAATTTCGTTATCGCTACTCGTTTCTTAATGCACTTAACTTCCGCTAAGTATTTTTCCATGCCGATAGTGTTTTTAAAAAGGCAATAAGATCGCATTTTAGGTTGGCTCTGTAAAGTCACCAATGTCTTTTGATTGTTACAATCAATTATTCGCTGGTATATGATAGCTGTCAGTTGACTCCTTGACGAGGTACTGTTATCCCTCGTATACCAAGGTTTCCTAGCCCATGTTTTTTCAATATATCCCTCAACAGTGATGTCCATAACGGGTTGCTAGCATCAAACTCAAGCCGTAACCAGTTTTTAATAGTTGCTTTAAATTTGTTGTATAACTTTATATTAAATGTTTGTAACAAAGTAAACACGTCATCAAGTAGTCAGCCTCTGTCATGTCATATTAACTGTGGTCATGTCATCTCCTGCCGTGTTCCTTGTCGGACAAAGCAGAAACGACGTAAAATGACGTCATACAGGACATTGCTATGGCTGGAAAAGAGGCTGTATCATTCAATTCAAATGAAAAGGAAGATAAACCATTAATTTTTCATGATGGCTTCTGTTTTGGGAAAGATTACTGTGTCTTTGAGTGAAGACGATCGCCTTTGACGATGGAGAGAAGAAGTCTTGCTCTCATTTGCTTCATTTATGCTCAACTTGGGTTAGAAGAAATTTCTGCTTTCAATATTACACATTTAAATGCAACGAAAGGTATGAATGATTTTCTTAATAATTGTCTTCACCGACACCAGTCGCAAAAAACAAAAGCCAGAGACGCACACACGAGAAAGGGAAACAACGACGGCTTCCTGTTTAATTTTGTCCATAAATAAAGCTTCCAAATTGATTATAACTTTCTTGATGAGGTTATTATGAGTCTGAAATATTTAATGAAATATTATCAATGCCTATAATAAATGAAGCACTAAAAGTGTTCAATATTCGATTGTTCCCTTTTCTCATTGCGCAAACTGAACTAGGCAACACAGACGTGTGTTTTTGTTCAAAATAATGGGTTTTTTTGTTTGTGATTAAAGTAGAAAGCAAAATTTCTGAATAAGTCATCGGTCAAGAGATAGGAAACATTTTTTACCAGGGGTTGTGGGAGGGATATCTATGATATATATGAAGGTGCCAATATGGCGTCCAAGCAAGGGTAAAGTATGAAAATATGCTCAAAACTCTTAACTCGTCTGCAAAATTATTACAGCAGATGGCATTTTTAGGATAAATTAAAATATATGGTCTGATTTATTCTAAATTCTTTTTGTAGGATTATTCATAAAAACTACTAAAGCAACTACAATAGAATCTTCAGCATGGTATGCAAAATTTAGTGGTATGAAATCACAAACAGTGATTGCTGCAGAAATGAGAGAAATTGAGACAACGGCACATTTGATATCAAGTGCAGTTCATGAGACAGTCATGCCAAGTTTGCAGAAAAGTGAAAGCAGCTCAAGGAATTTAATTTCTTCAATTGCTGATAAATCGAGCACAAACAAACAAATAGTCCAATCACGTTCAAAATTAGAATCATCATTTTCATCGTCATTATCATCATCATCATCTTCATCATCATCTTCATCATCATCATCATCATCATCATTATATACAATCTCTATCAAACACCATCAAAATCAGATGTCTTCTTCAATTTTCATACAAGCAACGCAAGTAACATCATTGCCAAATATAGATCTTTCTTACACAAAAAAGACAACTGATGAATTATATAGCATAACAAAAAGACCATCACTTACGCAAATGTCTGCTTCACCTGCTAATAGTACTTACTTTAGTAATTTTTCATCAATTTTAGGGGACACAAACATCACCATTCCTGTCGTGCAGATCATTGGAGTTTCTTCCACTTCTTTCATCAAGCTTGCTGCAACAATCACAAAAGAAGGGAAGATAACGCCATCATTATCTCTCAACCTCAAACATTCTCCAACAGAAACCATGAATTCTTCAGAAGCTATCAAATTAAAGACTTCATCATCAATTAATCTTCATCCTTCTGATAACATGTCATCCGTTTATGGAAACAAATCTCATAGTTCATTGAAGACAGTAACATCAGCTATCCGCTCTCCACAACCATCTGTTTTACTGAAAAATTCTGTCATCTCTGTAAACACTTCATCTTCCACATCAAAATTAATTAAAGATAATTCTACTATGTCACTGAACACAGAGTATTCCTCATCTGTCAATTTTGTTAATAAAATTTCCTTGCAACCAACTAGATACAAAACCAAATTCATAGTGGCTGCTTCTTCAACAGTTATGCCATCATCTTTAGGAGCTAATGCGAAGACAGTATCTCTTTCTTTTGACATGAGTACAGAGATATTCAAGTTGATACCAACGTCTATGGTTACTCCTTTAATAACGCAAGCAGTTGATGGAAGGACGACATCTTCAATTCATCCAACAAAAGTAGGAACAACAGATGGGAATATAGATTTAAAGAAAAAGAAACAAGAAAACAAAGGTAAGATATTTCATCTGTCTAAGGGGTAGTTTTAATCTTAATTTAATGAAAACATGGGGCATATATTTATAGCAGGAAATGTAAAATCACAACAGATGGTGACACTAAAGATGATGACTGAGGAATTTTTTACCAAGATGCATTGTGTTTTTTAATCAAATTCTTATAGAACTGTTAAAGATTCTTAATTGGAACGCACTGCGTCTTTGTAAAAAGTTCCTCGGTGCCATAATTAGCGTTATCATGTATTGATGTATAATTGCTATATATATTACAGTACTTTCTTTCTAGAAGCAAAACTGTTTTCATAAACAAGTAACAGCAGCTGCATTATTTATTCGCAATGCCATGGATTCACCACAACTAATTTGCTCATTTTCCATTTACAACTTCTTTAGTCTTTAGCACATGTAGCATATTTACAATGACTTTCAAAAGAATATATACCACATATTGAAAGACAAACCTGTTGTACTTTATTTGTGAAAACACACTCCCTAAAAATGTTGATGGTATCGTCATCTTCTTCTTAATTGTATCTTAAGAATGTGCAAAAGTTTATTTGGACTTTTAATAATTTTTCAGGATTGCCTGCATGGATAGCAATTTTGTCTGCAATTGCTGTATTCATTGCCATTATGACAATTGCTGTTGTTACATTCAAGTACATAAGATGGTAAGTTCTTTTGCAGCATCACTTTAGGACAGAAGCTTTCAAAAATGACTTCTCACAAACAATTATAATCCTGACAACCTGCTTAATTTCTCATTTGAGTCGTTGCTGTAGCCTGCGCATGGTGGAGGAAAAAGAAAATTTCCAACAATTTTGGCTGTTTATTTGCAGGCTAATTCATGGTAGACTTGGGAAAATAAATTTGACTTACACAAAGTCTAACTGTGAAAGATATTATGTTAATGATACTGTCAGATAGATCTCCAATTCACCTTTGGACTGAACTACTGCCTAATTTTGTTGTTCTGGCTAACCATAAGATGAACGAGCAATTGCCACCTTTCTGCAAGGGTAAATCCAATCTGGGTATTATCTTGCATTTGTGTATTGCAAAGGATCAGTGTGGATGGGACCAAGTATTACTGCGTGATTTACCATGGGAGTCAGAAAAATGTATCCACTTTTTGAAAAATCATAAATAAAAACTATGAAATTGATCAAAAAACATCAACTCTATTGTCTGACTTATAATTTACAATGCATGATTTTTCTCTCAAATAAAGAACTTACTGAAAATTGGTTTCACTGGGCTTTGGTTAAATAAGACTTGGATGGCTTTACATAATTCACTTTCTAACTATTAGAATGGAAAAAGATTGTTTTTTGGAATAGTCTTTGCATTATAATAACATGGAAACTGATAAAACGTATACAGGGTGTTCCAAAAAGCTGTACCCCATTTGTGCGGTTGCTGTGGAGGAGCTGTAACTTCTATTGCCCGGGTTTTTGGTACAATTGCATAGAATATGCTTCAACTTAGAGTTTGAGACCTTATTTTAGCTAATCGCAAAAGTGGTTGCTGATTTATGGCGCAAGAAAATCAAAAAAGTGAAAACTTCAAAAACAGCACTTTCTTTGTTCTCCAAAAATGTCAAAATCAAGTAAATATTGCAAGAAAATGCAATGGGTTAATGGTGTTGTGTTATCAGTAAACAATACAATTAACCCTTTATAAACTTTAGAAAAAATTCTAGTACAGCTCTAGAACCCTTTCTTGCAGTATTTACTTGATTTTGGCATTTTTGGAGAACAAAGAAAGTGCTGTTTTTGAAGTTTTTACTTTTTTTACTTTCTTGCGCCATAAATCAGCAGCCACTTGTCCGATTGGCTAGAACAAGGTCTCAAACTCTAAGTTGAAGTATATCCAATGCAACTGTATCAACAACAGGACAATAGAAGTTATAGCTCCACTGCAGCAGCCGCACAAATGGGCACAAGTTTTTTGGGACACCCTGCATATTTCCTCCAATCAAAGCTACAATCCTGGCCAAAAAAAGTTGGGACATAGGCAAATTTCAGTGTTGTTGTAATGTTTCCTAAAATATCGAGGCGAGAACGTACGTATAGGACCTTCTCCCTCCCCCCTAATTCAATGTTGCCAAGGTCAAGTGATTCAAGCAACATTGAAATGGGGGGAGCGGGGGCATGGAATCAAAGGCCAAAGTAGTGCAATTTTGTGATTTGTCCCAAGTATTTTGACCAAGATTGCAGGGACAAAAAAGAAAGTTTGTGTCAAATATCTATATTAGATTGAACAGCACATTTTTGATTGCTTTTAAGCAATTCAAGGAAAGTGGTTTTGGTGAGGTTGCTAATCTGAGGCGTTCAGACATTGCTTTTCACCGTAGCTACCTAAAAATATTCGGTTCCGCGTGATTTCTGCGTTATTGGCCTGGCCGTATTGGTAGCTAAAACTGGGTCGCTTTCGTGCCCTTACGTCATTCTTAGCAAGTATATCGAGTTAGCTGGGATTAACAATTCTTCCCTAGAATATTTGTTACGAGGTCTTACCTTTTTCGAGGAGCAAAATGTGCATAAATTACGTGTATCCAATGAGCCAATCTCATAGAGCCGAGCAAGGGAAATAGTGTTAGATGCTTTCGCTAAAATTGACCTAGACAAAAAGGTATTTTGTCTCTACAGTCTCCGAGTGAGAGGGGCAACGGCTGCCGCGAACACAGGTATAAGGGACAGACTTTTCAAGAGACATGGGAGATGGAAATCAGAAAATGCAAAGGATGGATACGTCAAGGACAAAACAAGCAGTCTTCTCTCTGTTTCTCTTAGTTTGGGAATTTAAACAAAAAACGCATTCCGTTTACCACCTGCAACATCATTGATGATCTTGGCTGTGTATGACGACCTCGCCAATTCACCTAAAACGGCTCTTTTTTGTCTTAATTCTCCGGAAAATTAAATAGGAAATAATTTATTTTCTTTTCCACCACAAAGTGGTGGATTAGAAAATAAAGATATTTACTAATGTTTTTGTAGGAAATTGTTATTGTACAATACAGTAATAGCCAACTTTTGAGAGAAGTAAAGCACCCAAAACTATGAGAAAATATCAAAGATTAAGTTGAAGCCCCATTGAGATCAAGATTGAGATTGAGATTGAGATTGAGAATGTTTAAATTATATGAATTGGTTGCATAGTTTTTGCAAGATGATTTTTCAAACAGTCGATACATTTTTTTAGTCAACCGGTATAAAATATCTGTCTGATGCACGTCTGTCTAATGTGGGTGCAGCTTTACTCTTGGTTGCTGTTGATAATTTGCTGATGCTACAGTTCCAAAGAACTTACATTATATAACGATGTGATATGAGGTGATTTTAATCTTGGAAGAAATCAATTATTTATTAGCAGTTGAAAAGATTTATTGTTATATAACTTTAGATATCATCAGATATAGCTCTTTATGCTATTTCTTTTTAAAAATTGATGAACAAACATGGTTTGACTTGGTTATATTGAGTGATGTTTTTCTTATCTTCATTAAAATTAATATTTCAGGAATAAAGACCATGGTGATTACACTCTATGGATTTCAAATTTGGAATTACAACCAATAACGTCAAGGTAATGGGACACCTTCTTCTCATTTTGTTACGTTGTTACTAAGAAACATTTATATTAACAACCGAATATTTTGGTGGGACAAAGTTTCAGAAAAATGCTTGTCGGAAATCTTATACTTATTTTACTGTAGTTATCATGGCTCGGAAATTGATTATGATCTACTAGCCAATTTAAAGACATTTTTAGGCTGGCCAAGCTGCCTTTTGTTCCACTTTTATGATATTCTGAATGTTTGTCTCTAGTTTTGCGTTGCCATTGTCTTATGTATTGTTAGTGGTGCTTTTAAAAATAATTTTCTGCTTCTTTTTGTTTCTTTAGGAATGCAGAGGTACTAACATAGGAATAAGACAATGAGGAATGAAACACAATGGATAAGTCTAGAATTTAGTGTAAATTTGAATCTTTAATTTATTAACAGCAGTGCAAGCCACAGCATACATGACACATTTAGAAAATATTCACGTTGTTCATTTGTATCAAGGGTTAAATATTGTTTGTATGTAAATAGTGAGGCTTTTGGTTCACCTTTCAATTTGGGAATATGGCAAAGTTATTTTGGTCTCAAGACACCATGTTTATATATTTCATTCATAGTCTATTCACTTTGTAGTTGTTATTTTGTTTCGTTGCTTCTTAGTGTTATTTATTAGACTTTGTTGTTATTTAAATTCACTTTTATTTATTCATGCAATCATTATAATATTCATAAAATTTCTCTGTAAGGCACATTTTTACATCAAAGGAACCAAATCCTATGTACCAAATTACTAGCATTCAAAACATAACAGCAAGATAACGATTTATATCTCTATACAGTTTATATCATGTCAAGACAATTATTAATAGCTGTATTTTGGAAGTATTCCTTTAAAACTTCATGTTTGAATTAACTGTTTTTCGATTCGTTGATCAAGTTACTAATGTATTTATTGAGTATAATCAGAGATAAGTTCTCAGTCTCTATTCTGTTTTCTTTAGAATCAAATGATAATAGTTTTATAAAAACTCGAACAAGATAACTTTTCACCAGATATCAAGCCTATAGTGTCACACTTTTGCCCAAAAATATCAGGGGCAATTTCTAGTCAAAAAGCAAGCCTCTAGTCTTAAATTTTTGCTCAATAATATAAACCCATAAGAATTAAGATCTATTTTCCCCACAAACAAGGCATTTTTTGAAGAAATCAAGGATTTCAAAATCAGCTAATTTTACCGAAAAGGCAAAGCCTATAACCTTACTTTTTCGCCGAAAAAGAGGATTTTCAATATCGGCCGATTTTACCGAAAAGCAAAGCCTATAGCCTTACTTTCTGGCCAAAAATAAGGATTTTCAAAATCTGCTAATTTTACCAAAAAAGCAAAGCCTATAGCCTTACTTTTTAGCCGAAAATAAGAATTTTCAAAATCGGCTAATTTTACCGAAAAAGCAAAGCCTATAGCCTTACTTTTTAGACGAAAACAAGGATTTTCAATATCGGCCGATTTTACCGAAAAAGCAAAGCCTATAGCCTTACTTTTTAGCCGAAAATCATGGCTTTCAAAATCAGCTAATTTTACCAAAAAAGCAAAGCCTTGTGTTTTGCCCAAAAATATGTGGGTTGACGTTTTCTCAAAATGCAAGTCTATTGCAAGTTAGTAATGACAAGTCTTCAAAGATTGCTAGAAAGCGTTAGCTCTTTACTCTAGGAGACATTTTCCCCTCACCCAGTAGATACATAGGAATCCTGGTCGGGGAGAAATTTTCAAGAAGATGACAGTGAAAATCTTAAATAGTAATAACAAGTATTTAAGGATTTCTGTTGTTTTAGCAATAAAAAATCTGCTATGTTACAGTGTCAGGATCAGCATTATTTTGCTGTCTTTTAATGTTTACAAGTGCTAATAACATCACAAGCGGCAAATGCAATAAACTAAATCTAAATAGTTTTCTGGATGAATTATTGTTTGAATCTTGATAAAACTTCCAGGCAAGAAAAGTCAAGTACAAGTTAATAGGTGTCACCTCGAGCATGAACCACCAATTTGTCACATCACACAAGGGAAGCATGAGAGCCAATGGGATTTGTGCTATACTATGGCGTAATGTGACTCGTCTACAAAGGGCTGGGTCGGTGACTGCCATCATTCTGTAACCAGCTGAAAAGAAAATAAGGACACTTAAGATCACGTGGTAGAAAGAGATTGAAATCAAACAGCATGAGTTACTACCAAATTATTAAATAAATATAATACAAAGAGGAGGATAACAAGATTTCGCTGTCTGTTGCAGTACAGTATAAAGCGTGTTAGTTTGAAAGGCTAGCCTAGGTTTCAAGTTGTATGAGACTGTCAATATATCAACATAGCGATAATAATCTATATTTTCTGTCTTAAAACTTCTGATATTGTCCGGTATATTCAAAACAATTCATATCAATGATAAAGAAAATTGCTTAATCACGTGACCACAGGTTAATCATCAAATATCTTTGCTTGAAGAAGTTTACGAAATTCCATTTCAAATTGACGTATTTGTGATTTCCTAAAAGAAGGCAGACCTGGAACTGGTTGGATTGGCACACCAGGAGAGTAAGTTTGTCCTTGCTGTCAGGAACAGTATGAATGTTGTTTTGTTTTCATAGCTCCCTGACAATGAGGATGGTGTTTCAATGTTTAAAAGCATTCCATACCTCTAGAGTAATCGGGCCTATAGCTCCAACTAAGTGCATTGAAATGTGGAAATTGCCAACAATACAGAATAGCAGCCAAGACCCAAGCACCAACATCTAGATTCCCTGTACATGCTGCCCATCCCATCAATGGAGGAATTCCTCCAACTACCGAACCAATCCAAGTATTAGCTATTGACACTCTTTTAGAAGGGGTGTAAATCCCAGCATATAAGGCTATATTCAGAGCACCTGAAAGAAAAGTAGTATCAAAGGTTAAACAGAAAGATTGCATTGAAAGATTGCAACAGAAAGAAATTAAATACAAATATATCAGAGAGCCAAAATATCGAATGTATTATGTTCCAATTAATTATCTTATTTATAAAAGCATTTTAGTCCCTACAAAGATTCGTACTTCTCCTAACTAAATTTCGAAACTATGAGAAATTGACTGTCAAAATAAGTGACAAACATAAATAAGATATACGTTGGACATTATTATCAATCATTTTACACCAATTAAAACAAAAGAATTAAGTATAGTGCTAGACTGTGGATATCAATTAAAATCATCAAGAAAGGAAACTGTAAAAGGGATACCAAATCAGCCTCATGCTGCCAAAAACTTGTTCTTCTTCACCAAAAATATCTTGAAAGCAAATACTATCTACAAAATAATTACCAACCTAAAACAGCAGTTAGTGAATTCGTACAAAGTCCAAGAATTGCAATACCAGTTAAACCAGCTACGCTTGCAAATAAGAATGCTTCTTGTGGCCTAAAAGGAATTAAGAACATTAAATTTAAATCTTGAAGAGTTTCTTTCAAAAATAAAAAACCATCAATAGTAACTAGGAACTGAGAGAGCATAGACTCTTTTTGAAGAAATCTTTAATTTACAAAAATTTTGTAGCTGCTTTTTGTGTGATATTTTCAAATAAAAATCAAAAAGGTTTAATTCAGAGCAACTAGTTGAGCATTTGTTGTTTTTAGATAACTTGTTGTTTTTAGATAACTTGTTGTTTTTAGATAACTTGTTGTTTTTAGATAGTTATGAGCTACAAATTAGTGACAATGCTGAAACACAAAAACAAAACAAAATAAAGAAGATTTAATAAATGATACGTACTGCAGTAAACCTTGAACTAAGACACGATTTCTTGTTCTGGACATTTGAGAGTCAAATGGAACTTCGATAAACTAGGGAGAGAGAAGATATCGACATTCACTTTAACAAATCTTTTCCATGTTGCCCTCGTTCTCAAATATATGCTTTAAGAACTTTCAGATAAATCAAAGAATTGTTTGCAATATACAAAGTTTAAATTTCAAAGAGACATTTTCAAAGACATTTTCAAAGTGACATTGTTTTTGGTTATCATCATACTAAAAGTTTTTCGTTATCTACATGTCTGGCTACTGCGAACTATTTAGAAAGCTCTTTAAAAATTGCCACTATCTGACAGATTGATGTACTAACAATGAAAAAGAAAGTGTGAACAATTCTTTGAGCAGTATATACATAACATAGAAATCATATTGAGCAACATAAATATTTACTCAATGGTTTTGGCAACAACCAGTGTAGTGCCTGGCAATACCGTATGGCAAACAGTTTACATTATGACACTCGATGTTTAAGGAATAAGGCAAACAAACCTGATTGAACGAATTAGCAGCACCAGAACATAAAGATGTCCCTATTGTCAGGCAAGTGAATGAATAAACACTGAAAGGGTCTGGTGCTAGAGCATATCCTGCCATTGCGGACAGAACTACCAGGCCTACAAAATATAAAATATGGAAAGCTATGTTCTAAGACAGTCAAAACAAAGGAAATAAAGATTAAAGGATCTATCTTGCATTTAGAAGTTAATACTTTATTTATACACTATCTAACAAGTCAGCACCCTGGAATTTTGGATTATTTCACTAACCCTGTAAAGAGATTACCTTATTTTTTGATTTTACAGGATTTTCAATTCAGTTTATTAAAGTCTCAAATTAAGTAATAACAAAATGCCACAATGAACACAAGGAAGAAGATGCCTATGTGAAAAAAATGTAATAAAAACTTATCAAGATCTTTTGTTGTGTCATTATTTTGTTTCTTCTCCTTGCATTATTTTGCTTCTTCTCAAATCCCAACAAATGTCCATGCTTTAAAATGAGCCATTCATAAGAAGTTTGTGCAAGAAAGTACAAGGGACAGCAAATCATCGACATTTATTTGGATTTGACAAGCAGCAAAACAAGCCAAGAAGGGGGTGTCCTTTTCTTGTTATGACCACACTTCTTAAGCATTTACCGGTGAAGAACATATTCATACACAGACCAGAAAATATAACAAATTTTCTTTTATTAAGATTTTTGCTTATTTGAAGAAATTCTACTAACTTAGTGACAGCCATGAGAAATCTACTGCAAAATAGTTAAGATACCTGATAATTTAGCTTTAGACAGTCTGAGGTAATATCCAATTTTGGTTTTCACATCAGCATTTGCAACCACCCATTTGCCTTCTTCCTTATCTAATGTTGCTGTTGTTTTGTCATCTGCAAAACCCCTTCTCATCTAAAAAAGAAACAGGCTTGTTTCCATTAATTTGGTGACAAAAATTTATTGAACTCAAAAAGCATTCCTCATGTCTTTGAATTAGTAAATCTGATATTGAAAAGATTTCAGGGCAATTTTTCCTCAAATCACGGTAAAACTAGAGATGGTTGGAAGGAATATTTTACCATGATCCTTTTTGTCACATAGAATTTAACATGGATTTGATTGGTAGATTTGACAGTGAATGCAAAAGATTGTGGTAACAAAATCCTTGATGTTGTCTTTGGCATAATAAGCAAAATTTTTTTGAAGGCAAAGTATACATAGTACCCAGGGTGTTTGAAGTTTTTTAAACATAAAGAACCACTGAAATTACATTATGTTGGCTGTTGATACAGTGGATTTTTCTTTAATCTTTGGAGAACTAGGCCAGCTTTAACAAAAAATAATAGTTGTTTGACAATGATCAACTCATTTAGATGTGCCCTTTAGAAAGAACAACTTTGTAAGAAAACTATAATCTAGACAATAAATATATTTTTTAACTAACATACCAATGAATATTTGAATTTTTTGGGAAAATCCAAATGTTGAGTTTTGTTCCATGGATAGTTGATCAAAGATGATCCAGTGTGTCTGATTTTCATGAAATTTGGTCTCAATGCCAAGTCTGGAAAAAGGCAATCATTTTCAAAGCAAAAGACAAACAATTTGCAACTTGATTAAGTTATCCTTAAATTTCCTATTTTTATCTTTAGATTTTTAACTATTGTTTTCTTAGCTTATGTCTTAGAATGTCAAAACTTTTCTGTCATAAGACTTATGTCCTGAAATTCTTTAATCACTCGCTCACTGAAACACAACACTTCAATTAATTATGCATTTACAGTACTGGGCTTCTTTGGTTGGACATAGCCACCATATAGGCATTCACTGCTAGTAGAGTGCTGTTATGTTATATTTATATACTTTATGCACTTTCCACATCGTAAGATGGCCACTGTGTTAAATGGTAGGCTAAGCTTAGCATCATGAAGTACACCTGTTCCTCGCTTTTATTGCAATTGCATAACTTTTCACGTTACGTTGTTCCCTTTTTTAGGCTTTTTATACCCAAACAAAAATGGCTGCCACACAGCACAGTTCATTTCATTAATACCTGTTGGACAGCGGCATCTTATTATCAGGTTAATCATCGCCAATCACGTTTGTGTCTTTACATTCTGAATCGATTTTTCCTCCGTTGTTTTAGTTTCTTCAATTCAAGCTTCCAATAATGATTTGAAACAGATTTTCTGATCAGTTTTCATGGCCTTCTCACCTTGATGTCTGTCCCATGTTTAGGCACCATATTAGATTGGCCTCTTATCCATGTCTAGCCCCCCTTTCAAGTTTTAGAATTTGCTTCCAAATAATTATGCGCATAGGGTGTCCATAGTTTTCATAAATCTACACAAGGAAGTGAAACCACGGAAATGAGCACACGAGCATAGGCATCAAATAGTGATTGACGAACAAATTGACATGTGACCATGAAATGAACCAATCACAATGTGCAAAATAACAACAAATCCGAATTTACGCTTCTTTCGCCTATCAACATCCTTCGCAATACCATTTCCTGCTGTACGGAGATTTAGCTCAACAATGCATCCCAAGATTTGGAAAAACTACGAATTGCGGTTTACCTGTTGGGTAGCAAATAAAATAAAACATAGAGCAATATAAAATAAAACAACAAACATAAGAGAACATAAAGGAATAGGTGTATTCAGTTCTCTCTTGTGACTATGGTTCGAGGAAGCGTGGTAAAATTCAGTAGTTTTAGCAATCAACTTCTCTCATATCTATGTATACAAAAACTAAAGAAAAATCTAGAAACAAATCTCAAAATAGCTTTTCTGAGTCAGTTCTTGTTCTATCCATAACTTTTTCGCTTTTTCCTAATTCCAACGCAATCCAAGAAAGATTCGTGAGCAAGATTGTAAATTTTCTAGAAAGTTCCATTGGTAATAACACGGAATTTCTAGAACATTCCACGAGGTCCAAGAAATTCTAGAAAGGTCTTTAGTCGCTATGTCAACAAAATAGTATGTTCCAGAATATTCCGACGTTTTGTAGAGGTTTTCGGTTTCTTCTAGAAGATTCTTTCAAAAAATGTGGAATTTTCGTGTCACTCGTGACATCTTTTTAATCAAATATTAAGTGCCTCAAAGAAAACAGACTTTGAACTTGGAGGAATTATCACCCATACAAGAAGGGGAATTTTGTGTGGCTGATTTTGTAATTTTGATAAATTTATCAATTACGTTTTGTACATTGATACTTTGAACTCCAAAACGATTACTGGACATTTAAAAAGAGGGGAATCTGAAAAGATTTACTGTTTCCCTTTGGTACACATATGGCACATAGTGGCACATATTGTCCACTATAGATTGCAGGATTTCAAACATCCAGTCAGCGACATAGTACCAACAAAAATTAATTGTTCTAGCAGGGTAAATCCGCGGTTCATCCGCGAGTAAATCTGCAGAATTCAATCAAACATTGATGCTCATATTTTTACTTGTTTCGATTTGATTAATTTAACTAGGATTTTATGATTTCCCTGCAACCTGTGCCAGACCAAAACAGCAGCATATTTCAACACGTTGGTACGCTCTCATCCAAGCATGTCTTTATGTCTTTGTGGCTATATCCACTCTTTCGCTTAAAACGCTGTTCTATAAGATGACATCTAGGCAAGGCGGATAGTGGAAATAATCGGTTCCCCTTTCAAAGGTGATTGGTTTTACGCCGCTTTCAACTTAAATGGAACAGTCCTCGAATGGCCAAACAATTTGATCTGTAACAAGCTTATGAGGAGAGAATAAACCTGGATAATGATTTCTTATTTATTAAAAGCTTTTTATTATTCCCCTCCTCGCCTCATATTTATATTGTTATGACTTACAGAGACAATTGCGCAGTTGTCTTTCCGTAGGCACACCTGCCACTCGTGTTCGGTGTTTAATAACAAAGTTTCGATCTTTCATGGGCCTTTCAAGTATCTCTTTGCACACAAATCCACTTGTGCCCAATGACACACATTACATCATATGACCGAAGAGATCATTGACCTGGACATTTTTCAGCGAACTAAAATCATTTCTGTTCCGAGAGACAGAACTAAAGTTCCCTCTGCTGTAAGGGGGAGTCCCCTTACTTGACAAGCTGCGACCACGCTATAAAAAGAGAGACTTTATTACCTGGGTTGTGATAAAGACGCTGGCGAAGTTTCAGAGGTGGGAAAACGAAGACAGAGTTACATTGGAACAGTGGCAAGTTGCAAAGGTACATTTTCATTTAATATTCAATTTTCTTTCTTGAGATTATATTGATTTGATGAAAGTTTGTAGGAAATAAAATCTGATATATGTTACCAGTCGTCGCTTTCCGTTAATTTACAATCTTTATTCAAGTCTAAGATTTCTACCTCAGATTTCTCTCAGGTTGCATTTCAAATCCTTTTCAAATCTACCTTCAAGATATCTAGGATAACAAATCATACGCAATAACTTAGTCTAATTCTTGTTTATTCAGAAGAACTGCTGCAGGCTAATTTCGAGACAGTCACGTGCAAATAAAGGAAGCGAAAAGGCTTTTATTAAAGAGCGCGTTGATGCTTAAATGAAATAAACAAATATTCCTGCGAGAAAAGAAAACTTAGACAGCTGAATGGAAAAAAGATCAATTCAAAAACAAGGTTATTTACGAAAAGAATCAGAAAAGTATTGTCATCCCCTTTACAAGTACCCGTTTAAACCTGAATATTGTCGTCAATGAAATCAATCATGTCTTTGGTGTTCATATTATGTTGCCTCGTTTTCGCACTCGCTTGTTGTTTGTCATCATTTTGCTATCGAAAGAGTCTTAGGTGCTTCTCTTTTGACGCTTATTCTGTTGTCTTCTTGCAGGTTAAAACAAAGAAGCGAATATATCTCTTTTGAAGACAAACAATTCACTCTCTTGAGAAGAATTGATTGACGCTGGCAAGTAGAGCACCAGAATGGCGGATAAAGGTGATTGTGATAAACCTTTTTGATCATTTCCTAGTTACTTATACCAGTATAATTTAACATGAGCTATTTCAAAACGCACGTTCTCTGCTATTCAAGTTAAGTTTGACATTCACTATCTTATCTATAGCTCAGATTGGTGGACACTGATATTCTACTCAAAACTAACAGTTGGTAGAAGCATTTCTAACAACGAATGACTTGCATCAAAACTTCCTATGTCCTTATACCAGTACAATTTAATAGATATTAGTTTTCGCTTGCACAATTAACATTTTTGTGTGACTACATGAGATAGAACATATTTTAAAGAATAGAGCCACCCCGTACATTATTCTTGCAGCCTTAAATAGACTTTCTCGATAATGTGCTATTTTCTCGTCCAAATTGGTAGTTTCACACCAGCTATCTTATCTTTAGTGCAGAATAAATTATGTTCTTTAAATTCGTATACACAAACAAGATGAGGCATGGGAAGCCTATTGTCGTTTCTCCGAAAGAATGAATATGGTGACGTTTTAATACATGGTGCATACATTTCGACGAACATTTTGTCGTTTGACACGGAGATAAATCACGACTACGGAGGCGTATTCTAGTTGTATAGTTGTAGATGTTTGCGCGAGAGCTTCCATTCGACGAGACACGGAAAGCAGGATCTACAGTAGGTGGAAAACGTCGCGCAAAGTTGTTTAACAAATTGATGTTGCTTTTGGACCAACCAACCTGATCATTAACTCTCGTCACATTTTGCATCAAGTGACAGTCAATAGATAGTAGATTCGAGTAGAACGAGCCTCGCTTAGATCCCTAGAGATACATTTTGATAATCCATCTTGAGGTCGACTATGTCTGGTTAATTTTGGCTTCGTTTCTCTTGGAAATAAAACTCCTGTGGTTTTTCCTTCAAATTGTGCAAAGGGAAATTCCAAATTCACTCATACTTCCAATCCAATATGCTGATCATCTATTTCTAAGAACTTCTAGAATTAGATACTGTTTCCTACCGCATGAATGACAACCACTTCCACTTAAGAACGAAATAAATAAAAGGTTCACCTTTCATTCTGATAATCTTTAGGCCACGAATTCGAGATCATCGTGTCAACAAGTGGAAGATTTGCTGCAGGGACGGACGCTCGAGTTTTCATCAAAATAACTGGAACAGAAGGAGCGAGTGATGAAATTGAATTGAAAGATTCAACGAAAGGAAAAAAGATTTTCGAAGCAGGAAGGTAAGTCCACAATTTCTAAGTTCCAAGAATCATTCAAATCTAAGTAAGTTATCATTCATTAGAATAACGAACATTCTGGCTTGGTATCTAATATTTTTGTTAACTAAGTCTATCAGTCTATCAGAATAGTTACCATAATCAATGTTCTGTTTTTTATGCAGAGAGATTACGAAATATTGCGTAATCTTAGTTTGTTTTTCACTTCTCCTGTTTCTACTCGTACTGATCTGTCTATTTATAGGGTTCCTTGTCCAATACTTGCACTGATACCAAACTAAAATCTGATTTCTCAAATAGCAACGACAAATTCGTGATCAAAGTCGACAAAAACATTGGTGTCATCATCAAGATCAGTATTCGTCATGATGATTCAGGGGCATTTTCTGGATGGCATCTTAATATGGTAAGATTTATTTTGATAAAGATATATATTTTCTCCTTCTCTCATTTACTCCTTTGCTCGTTTCTTCATTTCTTTTCCATTCCTTTCTGAAAGAATGTTAGCATTAAGTATTACTTTAAATTTGATGTGCGGTGCAGAAAAGCTGCGTGCAAGTTCATCGAATGTACCTTTTGTTTGAAGTTCCGGTGACCAAGATTGATATTTTAATTTACATCTTAAGCAACAAATGATAATACATGGCGTTTCCTTTCTATATGCATATATTTGTTTGCTTTCTTTAAGTGAACATTGTCTTTCCTAACAATTCTATATTTTCAGATGTGTAAGTAAATTAAAACACAATGCTTCAGCTTTCC
